# Dietary factors and risk for adverse pregnancy outcome: A Mendelian randomization analysis

**DOI:** 10.1002/fsn3.4412

**Published:** 2024-08-22

**Authors:** Fangxiang Mu, Lin Liu, Weijing Wang, Mei Wang, Fang Wang

**Affiliations:** ^1^ Department of Reproductive Medicine Lanzhou University Second Hospital Lanzhou Gansu China

**Keywords:** adverse pregnancy outcomes, dietary factor, Mendelian randomization

## Abstract

This study aims to explore the link between dietary habits and adverse pregnancy outcomes (APOs), including preterm birth (PB), preeclampsia (PE), gestational diabetes mellitus (GDM), fetal growth restriction (FGR), and spontaneous abortion (SA) through two‐sample Mendelian randomization (MR). We accessed publicly available genome‐wide association studies' (GWAS) summary statistics for dietary habits and APOs, respectively. We used five MR methods to synthesize MR estimates across genetic instruments. To ensure the robustness of our results, we assessed heterogeneity, and horizontal pleiotropy, and conducted sensitivity analyses. The primary analysis showed that intake of dried fruit (odds ratio (OR), 0.522; 95% confidence interval (CI): 0.291–0.935) and fresh fruit (OR, 0.487; 95% CI: 0.247–0.960) was related to a decreased risk of PB. While intake of tea (OR, 1.602; 95% CI: 1.069–2.403) and poultry (OR, 6.314; 95% CI: 1.266–31.488) was linked to a heightened risk of PB. Cheese intake was a protective factor against PE (OR, 0.557; 95% CI: 0.337–0.920) and GDM (OR, 0.391; 95% CI: 0.270–0.565). Intake of lamb/mutton had a negative relationship with PE (OR, 0.372; 95%CI: 0.145–0.954), whereas oily fish consumption showed a positive relationship with FGR (OR, 2.005; 95% CI: 1.205–3.339). However, after correction using the false discovery rate (FDR) analysis, only the intake of cheese showed a significant causal relationship with GDM (*p <* .001). Our study preliminarily found that cheese intake was significantly associated with the lower risk of GDM, while others were suggestively associated with the risk of APOs. Well‐designed prospective studies are still needed to confirm our findings in the future.

## Graphical abstract

This study used Mendelian randomization to investigate the relationship between dietary factors and adverse pregnancy outcomes (APOs). Cheese intake was significantly associated with a reduced risk of gestational diabetes mellitus, while other associations were suggestive. Further prospective studies are necessary to confirm these findings.

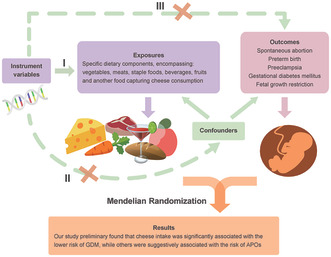

## 1 BACKGROUND

Adverse pregnancy outcomes (APOs) include preterm birth (PB), spontaneous abortion (SA), preeclampsia (PE), gestational diabetes mellitus (GDM), and fetal growth restriction (FGR). Globally, 11% of deliveries result in preterm births (<37 weeks of gestation) (Blencowe et al., 2012), 15.3% of recognized pregnancies end in SA (Quenby et al., 2021), 5%–8% of pregnancies are at risk of developing PE (>20 weeks of gestation) (“ACOG practice bulletin. Diagnosis and management of preeclampsia and eclampsia. Number 33, January 2002,” 2002; Khan et al., 2023), the prevalence of GDM is reported at 18% (Metzger et al., 2010), and 3%–6% of pregnancies face the threat of FGR (Hendrix et al., 2019).

Adverse pregnancy outcomes (APOs) bring great risks to both the mother and the developing fetus. For mothers, APOs such as GDM or PE not only elevate the risks of anxiety and depression but also increase the chances of cesarean section and complications in subsequent pregnancies (Kang et al., 2021; Kim et al., 2007; Roberts et al., 2022; Sibai et al., 2011). In the long term, mothers with conditions like GDM or PE may face higher risks of cardiovascular diseases (Osgood et al., 2011; Shah et al., 2008; Stuart et al., 2018). The fetus faces the risk of adverse outcomes, such as embryo‐stopping development, malformation, low birthweight or macrosomia, etc. (Brown et al., 2019; Joyce et al., 2021; Laisk et al., 2020; Loeken, 2020). In the long term, PB may have implications for a child's cognitive, behavioral, visual, and learning impairments (Filoche et al., 2018; Linsell et al., 2015). Additionally, GDM can heighten the offspring's risk of developing type 2 diabetes (T2D) later in life (Wicklow et al., 2018). Despite the considerable consequences of APOs, their exact etiology remains poorly understood. Therefore, it is necessary to identify modifiable risk or protective factors to prevent the onset and development of APOs.

Maternal nutrition during pregnancy is of paramount importance for pregnancy outcomes, birth outcomes, consequently, and the health of the offspring (Chia et al., 2019). Given that dietary factors are easy to obtain and change, there has been an increasing focus among researchers on their potential implications for APOs (Kukkonen et al., 2024; Li et al., 2024; Raghavan et al., 2019; Roberts et al., 2023; Sundermann et al., 2019). However, current research has not yielded consistent results. For example, one study has shown that greater adherence to a Mediterranean diet pattern (such as vegetables, legumes, fruits, nuts, and monounsaturated fats) around the time of conception was associated with reduced odds of developing any APO, particularly PE, and GDM, in a prospective cohort study of geographically, racially, and ethnically diverse nulliparous US women (Makarem et al., 2022). However, while some dietary habits exhibit protective effects during pregnancy, others have been linked to increased risks. A systematic review indicates that alcohol consumption during pregnancy is linked to a significant increase in miscarriage risk, with even moderate drinking elevating the risk by 6% for each additional drink consumed weekly (Sundermann et al., 2019). Several aspects of these kinds of studies should also be noted. First, most of this kind of research focuses on diet patterns, yet the precise relationship between specific dietary intakes and APOs remains unclear. While dietary pattern analysis provides a holistic view of the effects of multiple dietary components on diseases, it concurrently restricts the ability to explore the role of individual diets. Moreover, despite these prior observational studies controlled for various confounding factors, residual confounding remains inevitable.  A multitude of studies have established an association between diet and APOs, however, the causal relationship at the genetic level remains to be elucidated.

Mendelian randomization (MR) is an approach that uses genetic variants, which are randomly allocated at conception, as instrumental variables (IVs) to estimate the causal relationship between an exposure and an outcome. Since these genetic variants are determined at conception, they can minimize biases arising from confounding factors or reverse causation. In this study, we aim to elucidate the potential causal relationship between dietary habits and APOs. To achieve this, we undertook an MR investigation using summary data from genome‐wide association studies (GWAS).

## 2 METHODS

### 2.1 Data sources

The underlying assumptions for MR analysis include: (1) The instrumental variable (IV) must be strongly associated with the exposure. (2) The IV should not be directly related to the outcome. (3) The IV is not associated with any potential confounders (Sekula et al., 2016).

The GWAS summary statistics used in this study were provided by the Integrative Epidemiology Unit (IEU) OpenGWAS Project (https://gwas.mrcieu.ac.uk/). This project, supported by the MRC IEU at the University of Bristol, compiled and analyzed GWAS data from the UK Biobank, resulting in publications, as well as from the FinnGen Biobank. Ethical review was not required for this study since the data utilized were public, anonymous, and de‐identified.

In this investigation, we systematically evaluated specific dietary components, encompassing: vegetables (salads/raw and cooked types), meats (processed, poultry, beef, nonoily fish, oily fish, pork, and lamb/mutton variants), staple foods (bread and cereals), beverages (weekly alcoholic beverages, alcohol consumption frequency, tea, and coffee), fruits (dried and fresh), and another food capturing cheese consumption. These GWAS summary data were directly or indirectly extracted from the UK Biobank by the IEU OpenGWAS Project. Further details on the exposure datasets can be found in Table S1. Summary data from GWAS on SA (9113 cases and 89,340 controls), PB (5480 cases and 98,626 controls), PE (3903 cases and 114,735 controls), GDM (5687 cases and 123,579 controls), and FGR (2579 cases and 171,167 controls) were sourced from FinnGen. All participants in this study were of European descent.

### 2.2 Instrumental variable selection

In MR analysis, IVs function as intermediaries to discern potential causal relationships between exposures and outcomes. Typically, these IVs are characterized by genetic variations, with single‐nucleotide polymorphisms (SNPs) being predominantly employed. To ensure the relevance of these IVs to dietary factors, we sourced SNPs from the IEU OpenGWAS Project database. The criteria for SNP inclusion were stringent: a genome‐wide significance threshold of *p* < 5 × 10^−8^, a clumping window >10,000 kb, and a linkage disequilibrium of *r* ^2^ < .001. The robustness of the association between the selected IVs and the exposure was ascertained using the *F*‐statistic, with values exceeding 10 generally denoting a strong association. Comprehensive details regarding the selected SNPs are elaborated in Table S2.

### 2.3 Statistical analysis

**FIGURE 1:**
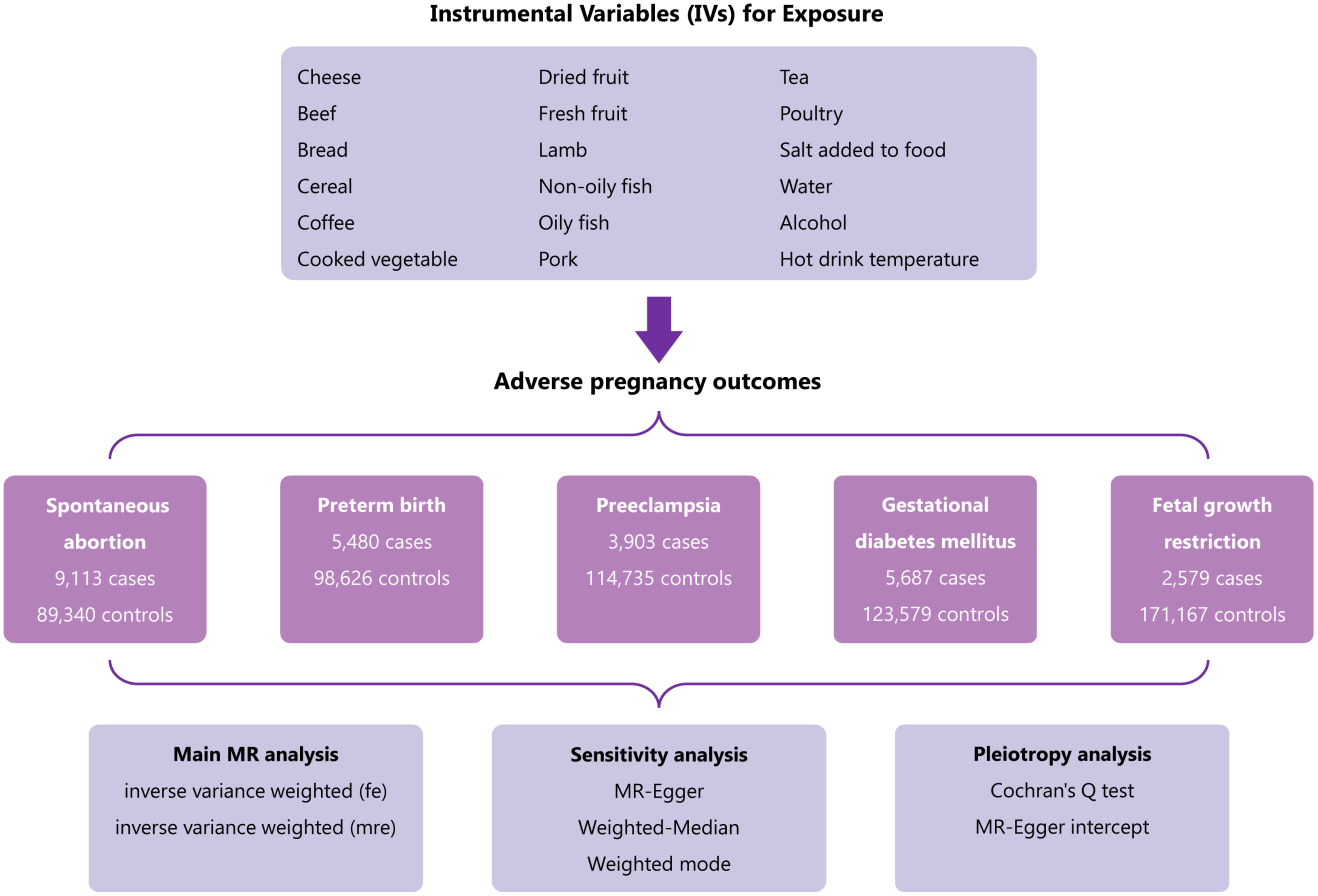
Flowchart.

The inverse‐variance weighted (IVW) method was employed as the primary method for estimating causal effects. The IVW model is the most powerful for detecting causal relations in two‐sample MR analysis. We contrasted the results of the IVW method with those from the weighted median (WM) and MR‐Egger methods. The WM method tolerates up to 50% invalid IVs, while the MR‐Egger method allows for all IVs to be invalid. Hence, consistency across all three methods is more convincing. The heterogeneity of the IVW model was assessed using Cochran's *Q* test, where *p* < .05 indicates heterogeneity. However, the presence of heterogeneity does not necessarily invalidate the IVW model. The MR‐Egger method accounts for potential horizontal pleiotropy by allowing a nonzero intercept. Leave‐one‐out analyses were performed to determine the influence of individual SNPs on the results (Figure 1). All analyses were conducted using the TwoSampleMR package in R software (version 4.2.0). The false discovery rate (FDR) method was applied to correct for multiple hypothesis testing, with *p* < .05 deemed statistically significant.

## 3 RESULTS

We examined the causal relationship between dietary factors and APOs using 18 different exposures. The number of SNPs (Nsnp) utilized in this study ranged from 6 to 58. All *F*‐statistics were above 10 (range: 29.740–493.643). For comprehensive details, please refer to Table S2.

**FIGURE 2:**
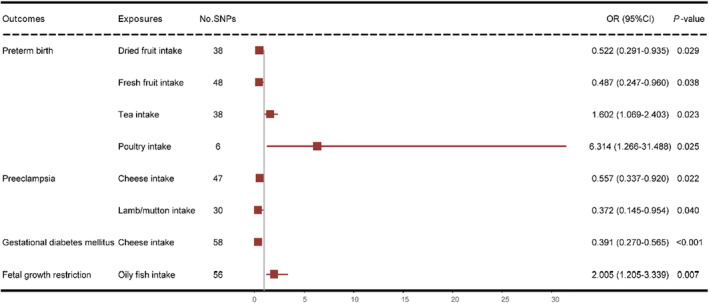
Forest plot showing results from Mendelian randomization study to assess associations between dietary intake and adverse pregnancy outcomes. CI, confidence interval; OR, odds ratio; SNPs, single‐nucleotide polymorphisms.

A total of eight causal associations were identified (IVW *p* < .05) (Figure 2, Table 1). We found an increased risk of PB associated with tea intake (OR: 1.602; 95% CI: 1.069–2.403; *p* = .023) and poultry intake (OR: 6.314; 95% CI: 1.266–31.488; *p* = .025). Intake of dried fruits (OR, 0.522; 95% CI: 0.291–0.935; *p* = .029) and fresh fruits (OR: 0.487; 95% CI: 0.247–0.960; *p* = .038) was identified as protective factors against PB. We observed protective effects against PE for cheese (OR: 0.557; 95% CI: 0.337–0.920; *p* = .022) and lamb/mutton consumption (OR: 0.372; 95% CI: 0.145–0.954; *p* = .040). Cheese intake was also found to be protective against GDM (OR: 0.391; 95% CI: 0.270–0.565; *p* < .001). Oily fish intake was associated with an increased risk of FGR (OR: 2.005; 95% CI: 1.205–3.339; *p* = .007). Following FDR correction, only the association between cheese intake and GDM remained significant (*p* < .001). Leave‐one‐out analyses demonstrated the robustness of the causal relationships (Figures S1–S4). Notably, no significant associations were observed between dietary factors and SA. Scatter plots of associations are presented in Figures S3 and S4.

**TABLE 1:** Mendelian randomization results of causal links between dietary factors and adverse pregnancy outcomes risk.

Outcomes	Exposures	Nsnp	Method	Beta	SE	OR (95%CI)	*p*‐Value	FDR	Heterogeneity		Horizontal pleiotropy		
									Cochran's *Q*	*p*‐Value	Egger intercept	SE	*p*‐Value
Preterm birth	Dried fruit intake	38	MR‐Egger	−0.661	1.430	0.516 (0.031–8.509)	.647		32.718	.670	0.000	0.017	.994
			WM	−0.322	0.414	0.725 (0.322–1.632)	.437						
			IVW	−0.651	0.298	0.522 (0.291–0.935)	**.029**	0.212					
			Weighted_mode	0.054	0.774	1.056 (0.232–4.810)	.945						
	Fresh fruit intake	48	MR‐Egger	−1.124	1.171	0.325 (0.033–3.227)	.342		41.271	.708	0.004	0.011	.719
			WM	−0.220	0.508	0.803 (0.297–2.173)	.665						
			IVW	−0.718	0.346	0.487 (0.247–0.960)	**.038**	0.212					
			Weighted_mode	−0.074	0.797	0.929 (0.195–4.433)	.927						
	Tea intake	38	MR‐Egger	0.655	0.454	1.925 (0.791–4.684)	.158		−0.004	.009	0.652	41.039	.298
			WM	0.574	0.299	1.776 (0.987–3.193)	.055						
			IVW	0.472	0.207	1.602 (1.069–2.403)	**.023**	0.139					
			Weighted_mode	0.678	0.369	1.970 (0.956–4.058)	.074						
	Poultry intake	6	MR‐Egger	−37.982	22.756	0.000 (0.000–750.226)	.170		5.148	.398	0.431	0.246	.155
			WM	2.579	1.000	13.179 (1.858–93.505)	.010						
			IVW	1.843	0.820	6.314 (1.266–31.488)	**.025**	0.139					
			Weighted_mode	3.021	1.557	20.505 (0.969–433.771)	.110						
Preeclampsia	Cheese intake	47	MR‐Egger	−1.279	1.139	0.278 (0.030–2.594)	.267		47.326	.418	0.012	0.019	.535
			WM	−0.688	0.381	0.503 (0.239–1.060)	.071						
			IVW	−0.586	0.256	0.557 (0.337–0.920)	**.022**	0.085					
			Weighted_mode	−0.854	0.813	0.426 (0.086–2.094)	.299						
	Lamb/mutton intake	30	MR‐Egger	−4.218	2.257	0.015 (0.000–1.228)	.072		37.042	.145	0.036	0.025	.152
			WM	−0.819	0.690	0.441 (0.114–1.704)	.235						
			IVW	−0.988	0.480	0.372 (0.145–0.954)	**.040**	0.316					
			Weighted_mode	−0.660	1.274	0.517 (0.043–6.269)	.608						
Gestational diabetes mellitus	Cheese intake	58	MR‐Egger	−1.455	0.965	0.233 (0.035–1.548)	.137		83.100	.014	0.009	0.016	.585
			WM	−0.919	0.284	0.399 (0.228–0.696)	.001						
			IVW	−0.940	0.188	0.391 (0.270–0.565)	**.000**	**0.000** *					
			Weighted_mode	−1.137	0.589	0.321 (0.101–1.018)	.059						
Fetal growth restriction	Oily fish intake	56	MR‐Egger	0.855	1.155	2.353 (0.245–22.631)	.462		58.450	.350	−0.002	0.017	.887
			WM	0.215	0.372	1.240 (0.598–2.569)	.563						
			IVW	0.696	0.260	2.005 (1.205–3.339)	**.007**	0.190					
			Weighted_mode	−0.068	0.716	0.935 (0.230–3.805)	.925						

Abbreviations: CI, confidence interval; FDR, false discovery rate; IVW, inverse‐variance weighted; Nsnp, number of SNPs; OR, odds ratio; SE, standard error; WM, weighted median.

Bold values denote significant *p* values for the IVW results.

* *p* value is still significant after multiple corrections.

## 4 DISCUSSION

In this two‐sample MR study, we characterized the association between 18 dietary intakes and the risk of APOs. We observed highly confident associations between cheese intake and GDM. Suggestive associations between dried fruit, fresh fruit, tea, poultry lamb/mutton, and oily fish intake and APOs were also detected.

Cheese, a fermented dairy delight, is consumed worldwide for its nutrient‐rich composition and easy digestibility. Cheese is rich in high‐quality protein, such as casein, lipids, minerals, and vitamins (e.g., vitamin A, B2, and folate), and probiotics and bioactive molecules (e.g., short‐chain fatty acids, milk fat globule membrane), which offers potential health advantages (Zhang et al., 2023). Extant literature suggests a potential association between dairy intake and PE or GDM risk. However, results concerning dairy products and GDM remain inconsistent. Evidence from observational studies delineates that dietary patterns marked by substantial consumption of high‐fat dairy products correlate with an augmented risk of GDM (Schoenaker et al., 2016). Predominantly, studies indicate no discernible linkage between dairy intake and GDM (Huang et al., 2023). Nonetheless, findings from our MR analysis robustly suggest a potent causal relationship between cheese intake and a diminished incidence of GDM. Our MR analysis may provide indirect evidence for the shared pathogenic mechanisms between GDM and T2D‐insulin resistance (Tieu et al., 2017). Moreover, in individuals diagnosed with GDM, as many as 50% advance to T2D over a five‐year period, reinforcing GDM's role as a predominant risk determinant for T2D (Bengtson et al., 2021). Consumption of low‐fat dairy products has garnered empirical support for its salutary effects against insulin resistance (Mishali et al., 2019; Sochol et al., 2019). A meta‐analysis concluded that increasing dairy and yogurt consumption by 200 and 50 grams a day was associated with 3% and 7% lower risks of T2D, respectively (Feng et al., 2022). Coincidentally, our MR investigation corroborates the protective role of cheese against PE, consistently with previous studies (Huang et al., 2023). Importantly, it should be noted that in our study, the categorization of cheese—which includes cheese found in pizzas, quiches, cheese sauce, etc.—did not differentiate between nonfat, low‐fat, and full‐fat types. As a result, interpretations of our findings should be approached with caution.

Possible mechanisms of dairy intake in preventing GDM and PE include the following: (1) Mitigation of insulin resistance: certain saturated fatty acids intrinsic to cheese may facilitate the consistent secretion of insulin (Slurink et al., 2022; Wai Linn et al., 2022). Concurrently, bioactive peptides engendered during the fermentation process might ameliorate pancreatic β‐cell function and augment insulin sensitivity (Li et al., 2021; Smith et al., 2023); (2) mineral homeostasis: cheese rich in calcium (Ca) and magnesium (Mg). These minerals play a pivotal role in preserving vascular homeostasis and curtailing muscular spasms. Supplemental calcium was shown to reduce PE risk by half in women with low calcium intake at baseline (Woo Kinshella et al., 2022); (3) antioxidative potency: constituent vitamins and antioxidants in cheese might potentially mitigate inflammation precipitated by oxidative duress (Barrera et al., 2015; Khan et al., 2019).

In our MR analysis, we observed suggestive evidence, indicating that both dried and fresh fruits confer a protective effect against PB. Numerous studies have demonstrated an association between fruit intake and a reduced incidence of PB (Zerfu et al., 2018). Fruits are rich in essential macro‐ and micronutrients, as well as health‐promoting bioactive compounds. Research has shown that fruit consumption can improve nutritional deficiencies and enhance dietary quality. Specifically, Myhre et al. found that dried fruit consumption, particularly raisins, reduced the risk of preterm prelabor rupture of membranes and was associated with an 18% decrease in the odds of spontaneous PB (Myhre et al., 2013). Similarly, Maldonado et al. reported that higher adherence to a dietary pattern rich in vegetables, oils, and fruit during late pregnancy was associated with a 69% reduction in the odds of PB (Maldonado et al., 2023).

In this study, tea intake emerged as a potential risk factor for PB. Consistently, an observational study by Chen et al. found that maternal caffeine intake from tea was associated with a 36% increased risk of PB (Chen et al., 2018). Tea primarily contains components, such as theophylline, polyphenols, and caffeine (Di Matteo et al., 2023). Caffeine might be associated with PB, and two potential mechanisms have been proposed by scholars. One hypothesis is that caffeine intake could elevate catecholamines, particularly epinephrine, leading to uteroplacental vasoconstriction and subsequent fetal hypoxia, which may impact fetal growth and development. Another hypothesis suggests that caffeine consumption could increase cellular cyclic adenosine monophosphate (cAMP) levels by inhibiting phosphodiesterase, the enzyme responsible for cAMP breakdown. cAMP buildup may then affect fetal growth by affecting cell division or by causing catecholamine‐mediated vasoconstriction (Chen et al., 2018).

Intriguingly, our findings suggest that while poultry intake may pose a potential risk for PB, consumption of lamb/mutton appears to offer a protective effect against PE. However, the underlying mechanisms remain elusive. Future investigations are warranted to elucidate the biological pathways and validate these findings in diverse populations.

Distinct from other meat products, oily fish is characterized by its unique fatty acid profile, with a particularly high proportion of unsaturated fatty acids. Consistent with previous observational studies, our study found nominal evidence of a link between oily fish intake and an increased FGR risk. A Danish study associated increased intake of oily fish with a heightened risk of FGR, a phenomenon they attributed to persistent pollutants found in fish from the Baltic Sea (Halldorsson et al., 2007). In a distinct study from the United States, fish consumption was inversely related to fetal growth, yet showed no correlation with the duration of gestation (Oken et al., 2004). Notably, this study only found the suggestive effects of dried fruit, fresh fruit, tea, poultry lamb/mutton, oily fish, and cereal on APOs. Considering the modest effect size, our results should be interpreted cautiously.

One of the strengths of this study lies in its utilization of MR to investigate the associations between diverse dietary intakes and APOs, making it one of the most comprehensive analyses characterizing the relationship between diet and APOs to date. Importantly, the MR design is inherently less susceptible to residual confounding. By employing multiple MR methodologies, leveraging the PhenoScanner database, and excluding SNPs associated with multiple dietary factors, we have effectively mitigated the potential influence of pleiotropy on our outcomes. Consequently, our results are less likely to be confounded by horizontal pleiotropy. Another significant advantage of this study is the sourcing of genetic variants related to dietary intake and APOs from summary‐level data of GWAS, which encompasses a large sample size.

This study has some limitations. Despite our rigorous control measures, there is potential for unmeasured confounding in the IVs sourced from the UK Biobank. The study hinges on the assumption of monotonicity for many IVs, which may be violated, possibly affecting the generalizability of our findings to broader populations. Our focus on specific food items overlooks the synergistic or antagonistic effects of habitual diets. Furthermore, the inability to conduct stratified analyses based on age, or specific dietary combinations, or to detect nonlinear associations, due to lack of detailed summary‐level data, restricts the depth of our findings. While MR helps mitigate biases from confounding and reverse causality, the results should be approached with caution until corroborated by well‐structured prospective studies.

## 5 CONCLUSIONS

In this study, we explored the associations between genetically predicted dietary intake and APOs. Our preliminary findings suggest that cheese consumption might significantly lower the risk of GDM. Moreover, dried fruit, fresh fruit, and lamb/mutton showed potential associations with a decreased risk of the same APOs. Conversely, tea, poultry, and oily fish intake demonstrated suggestive correlations with an increased risk of certain APOs. This study provides a foundation for future investigation of dietary intake for the prevention of APOs. It is crucial to approach these results with caution, and further validation through meticulously designed prospective studies is essential.

## AUTHOR CONTRIBUTIONS

**Fangxiang Mu:** Conceptualization (equal); writing – original draft (equal). **Lin Liu:** Data curation (equal); formal analysis (equal). **Weijing Wang:** Data curation (equal); formal analysis (equal); visualization (equal). **Mei Wang:** Visualization (equal). **Fang Wang:** Conceptualization (equal); writing – review and editing (equal).

## FUNDING INFORMATION

This study was funded by the Science Foundation of Lanzhou University Second Hospital (Grant No. YJS‐BD‐19), the Science Foundation of Lanzhou University (Grant No. 071100132), and the Medical Innovation and Development Project of Lanzhou University (Grant No. lzuyxcx‐2022‐137).

## CONFLICT OF INTEREST STATEMENT

The authors declare that they have no competing interests.

## Supporting information


Appendix S1.

## Data Availability

The datasets used and/or analyzed during the current study are available from the corresponding author upon reasonable request.
