# Supplementary material for: Dietary factors and risk for adverse pregnancy outcome: A Mendelian randomization analysis
Source: Food Sci Nutr. 2024 Aug 22;12(10):8150–8. doi: 10.1002/fsn3.4412 (PMC11521750; doi:10.1002/fsn3.4412)
Supplement: Supplementary file 1 — Appendix S1. [file FSN3-12-8150-s001.zip › fsn34412-sup-0006-TableS2.docx]

**Table S2.** SNPs used as instrumental variables from dietary factors and preterm birth GWASs

| **Traits** | **SNPs** | | **Effect** | | **Other** | | **Dietary factors** | | | | | | **Preterm birth** | | | | | | $\mathbf{R}^{\boldsymbol{2}}$ | | **F** | |
| --- | --- | --- | --- | --- | --- | --- | --- | --- | --- | --- | --- | --- | --- | --- | --- | --- | --- | --- | --- | --- | --- | --- |
|  |  |  | **allele** | | **allele** | | **Beta** | | **SE** | | ***P*-value** | | **Beta** | | **SE** | | ***P*-value** | |  |  |  |  |
| Dried fruit intake | rs10026792 | | A | | G | | 0.011 | | 1.84E-03 | | 3.90E-09 | | -0.002 | | 0.021 | | 0.911 | | 0.00008 | | 34.662 | |
|  | rs10129747 | | G | | A | | 0.009 | | 1.68E-03 | | 2.60E-08 | | 0.005 | | 0.020 | | 0.810 | | 0.00007 | | 30.988 | |
|  | rs10740991 | | C | | G | | 0.017 | | 1.86E-03 | | 2.00E-19 | | -0.004 | | 0.023 | | 0.862 | | 0.00019 | | 81.233 | |
|  | rs10896126 | | G | | A | | -0.015 | | 1.82E-03 | | 1.60E-16 | | 0.024 | | 0.024 | | 0.308 | | 0.00016 | | 68.068 | |
|  | rs11152349 | | A | | G | | 0.010 | | 1.82E-03 | | 4.90E-08 | | 0.002 | | 0.022 | | 0.916 | | 0.00007 | | 29.740 | |
|  | rs11586016 | | C | | G | | 0.010 | | 1.73E-03 | | 1.10E-08 | | -0.025 | | 0.021 | | 0.227 | | 0.00008 | | 32.590 | |
|  | rs11632215 | | C | | A | | -0.014 | | 2.58E-03 | | 4.40E-08 | | 0.016 | | 0.030 | | 0.592 | | 0.00007 | | 29.963 | |
|  | rs11772627 | | C | | G | | 0.018 | | 2.17E-03 | | 3.00E-17 | | 0.013 | | 0.022 | | 0.558 | | 0.00017 | | 71.316 | |
|  | rs11811826 | | A | | T | | 0.013 | | 2.01E-03 | | 4.40E-11 | | 0.012 | | 0.024 | | 0.600 | | 0.00010 | | 43.425 | |
|  | rs12137234 | | T | | C | | 0.010 | | 1.84E-03 | | 2.80E-08 | | 0.010 | | 0.022 | | 0.665 | | 0.00007 | | 30.847 | |
|  | rs1582322 | | G | | A | | 0.010 | | 1.72E-03 | | 6.80E-09 | | -0.035 | | 0.022 | | 0.106 | | 0.00008 | | 33.588 | |
|  | rs1622515 | | G | | A | | 0.010 | | 1.67E-03 | | 2.90E-09 | | -0.017 | | 0.020 | | 0.393 | | 0.00008 | | 35.232 | |
|  | rs1648404 | | T | | C | | 0.009 | | 1.67E-03 | | 1.80E-08 | | -0.028 | | 0.020 | | 0.158 | | 0.00008 | | 31.655 | |
|  | rs17175518 | | A | | C | | 0.011 | | 1.98E-03 | | 5.90E-09 | | -0.074 | | 0.026 | | 0.004 | | 0.00008 | | 33.877 | |
|  | rs17184707 | | T | | C | | -0.011 | | 2.04E-03 | | 2.10E-08 | | 0.025 | | 0.024 | | 0.302 | | 0.00007 | | 31.434 | |
|  | rs1797235 | | C | | G | | -0.010 | | 1.74E-03 | | 8.90E-09 | | 0.003 | | 0.021 | | 0.872 | | 0.00008 | | 33.076 | |
|  | rs2533273 | | A | | C | | -0.010 | | 1.68E-03 | | 3.90E-09 | | -0.004 | | 0.021 | | 0.857 | | 0.00008 | | 34.680 | |
|  | rs261809 | | G | | A | | -0.010 | | 1.68E-03 | | 9.80E-09 | | -0.020 | | 0.020 | | 0.317 | | 0.00008 | | 32.886 | |
|  | rs3101339 | | C | | A | | 0.014 | | 1.71E-03 | | 6.20E-17 | | 0.004 | | 0.021 | | 0.862 | | 0.00017 | | 69.907 | |
|  | rs34162196 | | T | | C | | -0.022 | | 2.77E-03 | | 7.10E-16 | | 0.025 | | 0.035 | | 0.473 | | 0.00015 | | 65.097 | |
|  | rs3764002 | | T | | C | | 0.013 | | 1.90E-03 | | 5.10E-12 | | -0.018 | | 0.021 | | 0.388 | | 0.00011 | | 47.653 | |
|  | rs4140799 | | A | | G | | 0.009 | | 1.68E-03 | | 1.80E-08 | | -0.003 | | 0.020 | | 0.882 | | 0.00008 | | 31.743 | |
|  | rs4149513 | | A | | G | | 0.012 | | 1.67E-03 | | 2.20E-12 | | 0.007 | | 0.020 | | 0.732 | | 0.00012 | | 49.254 | |
|  | rs4269101 | | G | | T | | -0.014 | | 1.86E-03 | | 1.10E-13 | | 0.055 | | 0.021 | | 0.009 | | 0.00013 | | 55.172 | |
|  | rs4800488 | | A | | C | | 0.012 | | 1.67E-03 | | 7.70E-13 | | -0.004 | | 0.020 | | 0.832 | | 0.00012 | | 51.368 | |
|  | rs57499472 | | C | | T | | 0.010 | | 1.72E-03 | | 8.10E-09 | | -0.005 | | 0.020 | | 0.820 | | 0.00008 | | 33.262 | |
|  | rs62084586 | | C | | T | | 0.013 | | 2.26E-03 | | 3.20E-09 | | -0.007 | | 0.025 | | 0.764 | | 0.00008 | | 35.073 | |
|  | rs6765212 | | T | | C | | 0.011 | | 1.89E-03 | | 5.60E-09 | | -0.007 | | 0.022 | | 0.762 | | 0.00008 | | 33.982 | |
|  | rs72720396 | | G | | A | | 0.011 | | 1.99E-03 | | 8.70E-09 | | 0.003 | | 0.027 | | 0.912 | | 0.00008 | | 33.123 | |
|  | rs75641275 | | C | | A | | -0.014 | | 2.39E-03 | | 2.90E-09 | | -0.009 | | 0.024 | | 0.718 | | 0.00008 | | 35.250 | |
|  | rs7582086 | | T | | G | | -0.010 | | 1.67E-03 | | 8.80E-09 | | 0.020 | | 0.020 | | 0.331 | | 0.00008 | | 33.088 | |
|  | rs7599488 | | T | | C | | -0.010 | | 1.69E-03 | | 6.70E-10 | | -0.028 | | 0.020 | | 0.161 | | 0.00009 | | 38.104 | |
|  | rs7808471 | | C | | T | | -0.012 | | 1.79E-03 | | 1.10E-10 | | -0.017 | | 0.021 | | 0.434 | | 0.00010 | | 41.720 | |
|  | rs7829800 | | G | | A | | -0.010 | | 1.79E-03 | | 5.10E-09 | | -0.021 | | 0.023 | | 0.352 | | 0.00008 | | 34.169 | |
|  | rs8081370 | | T | | C | | -0.017 | | 2.94E-03 | | 1.40E-08 | | 0.031 | | 0.028 | | 0.263 | | 0.00008 | | 32.181 | |
|  | rs862227 | | G | | A | | -0.009 | | 1.67E-03 | | 4.30E-08 | | 0.026 | | 0.020 | | 0.199 | | 0.00007 | | 30.030 | |
|  | rs893856 | | A | | G | | -0.013 | | 2.35E-03 | | 1.30E-08 | | 0.038 | | 0.026 | | 0.141 | | 0.00008 | | 32.346 | |
|  | rs9385269 | | T | | C | | 0.012 | | 1.68E-03 | | 7.20E-13 | | 0.000 | | 0.020 | | 0.983 | | 0.00012 | | 51.482 | |
| Fresh fruit intake | rs10064431 | | C | | T | | -0.008 | | 1.22E-03 | | 6.00E-10 | | -0.003 | | 0.020 | | 0.896 | | 0.00009 | | 38.311 | |
|  | rs10249294 | | A | | G | | 0.020 | | 1.26E-03 | | 4.10E-54 | | 0.002 | | 0.021 | | 0.923 | | 0.00054 | | 239.939 | |
|  | rs10271924 | | T | | C | | -0.007 | | 1.26E-03 | | 2.00E-08 | | -0.002 | | 0.021 | | 0.929 | | 0.00007 | | 31.481 | |
|  | rs1051547 | | C | | T | | -0.008 | | 1.24E-03 | | 1.10E-09 | | 0.013 | | 0.020 | | 0.514 | | 0.00008 | | 37.224 | |
|  | rs10828266 | | G | | A | | 0.012 | | 1.36E-03 | | 8.10E-20 | | -0.001 | | 0.022 | | 0.959 | | 0.00019 | | 83.023 | |
|  | rs10838724 | | T | | G | | 0.009 | | 1.28E-03 | | 2.10E-12 | | 0.010 | | 0.022 | | 0.646 | | 0.00011 | | 49.344 | |
|  | rs10840126 | | G | | A | | -0.008 | | 1.29E-03 | | 1.90E-09 | | -0.014 | | 0.021 | | 0.504 | | 0.00008 | | 36.028 | |
|  | rs11032362 | | A | | G | | 0.012 | | 2.12E-03 | | 5.30E-09 | | -0.036 | | 0.045 | | 0.416 | | 0.00008 | | 34.063 | |
|  | rs11085749 | | A | | G | | -0.008 | | 1.25E-03 | | 7.10E-10 | | -0.037 | | 0.021 | | 0.074 | | 0.00009 | | 37.987 | |
|  | rs111526888 | | G | | A | | 0.010 | | 1.36E-03 | | 3.70E-14 | | -0.051 | | 0.023 | | 0.025 | | 0.00013 | | 57.330 | |
|  | rs111915841 | | C | | G | | 0.008 | | 1.31E-03 | | 4.80E-09 | | -0.009 | | 0.022 | | 0.667 | | 0.00008 | | 34.267 | |
|  | rs11248509 | | T | | A | | 0.007 | | 1.27E-03 | | 7.40E-09 | | 0.010 | | 0.021 | | 0.627 | | 0.00007 | | 33.414 | |
|  | rs11896330 | | A | | G | | -0.008 | | 1.27E-03 | | 3.40E-11 | | 0.003 | | 0.022 | | 0.891 | | 0.00010 | | 43.919 | |
|  | rs12044599 | | G | | A | | 0.009 | | 1.50E-03 | | 3.70E-10 | | 0.009 | | 0.024 | | 0.702 | | 0.00009 | | 39.271 | |
|  | rs12536253 | | C | | G | | -0.008 | | 1.42E-03 | | 8.30E-09 | | 0.019 | | 0.026 | | 0.471 | | 0.00007 | | 33.205 | |
|  | rs12641371 | | T | | C | | 0.008 | | 1.23E-03 | | 1.40E-10 | | -0.031 | | 0.020 | | 0.124 | | 0.00009 | | 41.147 | |
|  | rs12780952 | | A | | G | | 0.007 | | 1.35E-03 | | 3.40E-08 | | -0.004 | | 0.022 | | 0.861 | | 0.00007 | | 30.492 | |
|  | rs12885598 | | A | | G | | 0.008 | | 1.25E-03 | | 1.70E-09 | | -0.002 | | 0.020 | | 0.937 | | 0.00008 | | 36.301 | |
|  | rs13072255 | | C | | A | | 0.009 | | 1.22E-03 | | 2.10E-13 | | -0.014 | | 0.021 | | 0.482 | | 0.00012 | | 53.867 | |
|  | rs1356292 | | T | | C | | 0.009 | | 1.55E-03 | | 3.50E-09 | | -0.032 | | 0.025 | | 0.206 | | 0.00008 | | 34.890 | |
|  | rs139042899 | | C | | A | | 0.036 | | 6.08E-03 | | 3.20E-09 | | -0.075 | | 0.079 | | 0.341 | | 0.00008 | | 35.037 | |
|  | rs149449 | | A | | G | | 0.007 | | 1.22E-03 | | 2.40E-09 | | 0.024 | | 0.021 | | 0.241 | | 0.00008 | | 35.655 | |
|  | rs1620977 | | G | | A | | -0.013 | | 1.38E-03 | | 1.10E-21 | | -0.001 | | 0.023 | | 0.963 | | 0.00020 | | 91.452 | |
|  | rs17049185 | | T | | G | | 0.008 | | 1.39E-03 | | 7.30E-09 | | -0.034 | | 0.023 | | 0.133 | | 0.00007 | | 33.444 | |
|  | rs1866823 | | A | | G | | 0.007 | | 1.24E-03 | | 2.10E-09 | | 0.031 | | 0.021 | | 0.137 | | 0.00008 | | 35.876 | |
|  | rs1964272 | | A | | G | | 0.008 | | 1.22E-03 | | 1.10E-11 | | -0.021 | | 0.020 | | 0.288 | | 0.00010 | | 46.132 | |
|  | rs2093654 | | G | | A | | 0.007 | | 1.26E-03 | | 1.50E-08 | | 0.020 | | 0.020 | | 0.323 | | 0.00007 | | 32.078 | |
|  | rs2143081 | | A | | G | | 0.008 | | 1.23E-03 | | 1.30E-11 | | 0.002 | | 0.020 | | 0.939 | | 0.00010 | | 45.844 | |
|  | rs2790688 | | T | | C | | 0.011 | | 1.70E-03 | | 1.50E-11 | | -0.012 | | 0.026 | | 0.654 | | 0.00010 | | 45.544 | |
|  | rs28479795 | | T | | C | | 0.011 | | 1.47E-03 | | 2.50E-14 | | 0.009 | | 0.023 | | 0.696 | | 0.00013 | | 58.113 | |
|  | rs2867113 | | A | | G | | -0.014 | | 1.96E-03 | | 1.50E-12 | | 0.046 | | 0.027 | | 0.091 | | 0.00011 | | 49.996 | |
|  | rs329274 | | G | | A | | 0.007 | | 1.23E-03 | | 2.80E-08 | | 0.004 | | 0.020 | | 0.824 | | 0.00007 | | 30.822 | |
|  | rs34162196 | | T | | C | | -0.018 | | 2.03E-03 | | 4.00E-19 | | 0.025 | | 0.035 | | 0.473 | | 0.00018 | | 79.890 | |
|  | rs4302893 | | A | | G | | 0.007 | | 1.30E-03 | | 1.30E-08 | | 0.002 | | 0.020 | | 0.910 | | 0.00007 | | 32.323 | |
|  | rs559734 | | C | | G | | 0.008 | | 1.36E-03 | | 1.10E-08 | | -0.021 | | 0.021 | | 0.307 | | 0.00007 | | 32.587 | |
|  | rs60452247 | | A | | G | | 0.008 | | 1.27E-03 | | 3.40E-10 | | 0.032 | | 0.022 | | 0.134 | | 0.00009 | | 39.422 | |
|  | rs6475724 | | T | | C | | 0.008 | | 1.37E-03 | | 1.90E-08 | | -0.022 | | 0.022 | | 0.313 | | 0.00007 | | 31.579 | |
|  | rs71386977 | | C | | G | | 0.010 | | 1.78E-03 | | 1.60E-08 | | -0.037 | | 0.030 | | 0.218 | | 0.00007 | | 31.937 | |
|  | rs739320 | | C | | T | | -0.009 | | 1.28E-03 | | 1.90E-12 | | 0.010 | | 0.021 | | 0.644 | | 0.00011 | | 49.635 | |
|  | rs7554485 | | C | | T | | -0.008 | | 1.25E-03 | | 1.70E-10 | | 0.010 | | 0.020 | | 0.630 | | 0.00009 | | 40.793 | |
|  | rs7818437 | | C | | T | | -0.008 | | 1.45E-03 | | 3.00E-08 | | 0.053 | | 0.028 | | 0.058 | | 0.00007 | | 30.701 | |
|  | rs7869969 | | G | | A | | 0.008 | | 1.30E-03 | | 5.70E-09 | | -0.027 | | 0.021 | | 0.187 | | 0.00008 | | 33.927 | |
|  | rs7982441 | | C | | T | | -0.008 | | 1.38E-03 | | 9.80E-10 | | -0.012 | | 0.024 | | 0.611 | | 0.00008 | | 37.371 | |
|  | rs8095324 | | G | | A | | -0.007 | | 1.25E-03 | | 2.70E-08 | | 0.013 | | 0.021 | | 0.550 | | 0.00007 | | 30.927 | |
|  | rs862227 | | G | | A | | -0.010 | | 1.22E-03 | | 1.10E-16 | | 0.026 | | 0.020 | | 0.199 | | 0.00015 | | 68.702 | |
|  | rs9517948 | | T | | C | | 0.007 | | 1.23E-03 | | 1.70E-08 | | -0.035 | | 0.020 | | 0.088 | | 0.00007 | | 31.807 | |
|  | rs9919429 | | G | | A | | -0.007 | | 1.22E-03 | | 3.80E-08 | | 0.006 | | 0.020 | | 0.749 | | 0.00007 | | 30.226 | |
|  | rs994270 | | G | | C | | 0.013 | | 1.44E-03 | | 4.20E-20 | | 0.017 | | 0.026 | | 0.502 | | 0.00019 | | 84.318 | |
| Tea intake | rs10741694 | | C | | T | | 0.015 | | 2.19E-03 | | 7.90E-12 | | -0.031 | | 0.023 | | 0.174 | | 0.00010 | | 46.784 | |
|  | rs10752269 | | A | | G | | -0.013 | | 2.12E-03 | | 1.30E-09 | | -0.024 | | 0.021 | | 0.254 | | 0.00008 | | 36.878 | |
|  | rs10764990 | | A | | G | | -0.012 | | 2.17E-03 | | 1.90E-08 | | 0.017 | | 0.020 | | 0.386 | | 0.00007 | | 31.589 | |
|  | rs11164870 | | G | | C | | -0.012 | | 2.18E-03 | | 4.20E-08 | | -0.025 | | 0.021 | | 0.223 | | 0.00007 | | 30.037 | |
|  | rs1156588 | | G | | A | | -0.015 | | 2.60E-03 | | 2.90E-09 | | 0.015 | | 0.024 | | 0.524 | | 0.00008 | | 35.241 | |
|  | rs11587444 | | G | | A | | 0.014 | | 2.17E-03 | | 1.00E-10 | | -0.020 | | 0.020 | | 0.319 | | 0.00009 | | 41.788 | |
|  | rs12591786 | | T | | C | | -0.018 | | 2.94E-03 | | 3.70E-10 | | -0.056 | | 0.034 | | 0.095 | | 0.00009 | | 39.274 | |
|  | rs13282783 | | T | | C | | -0.014 | | 2.35E-03 | | 7.90E-09 | | 0.028 | | 0.020 | | 0.168 | | 0.00007 | | 33.289 | |
|  | rs132904 | | C | | G | | 0.017 | | 2.55E-03 | | 7.80E-11 | | 0.005 | | 0.023 | | 0.845 | | 0.00009 | | 42.296 | |
|  | rs141071726 | | A | | G | | 0.041 | | 6.81E-03 | | 2.20E-09 | | -0.040 | | 0.086 | | 0.646 | | 0.00008 | | 35.753 | |
|  | rs1481012 | | G | | A | | -0.026 | | 3.36E-03 | | 5.30E-15 | | -0.018 | | 0.038 | | 0.647 | | 0.00014 | | 61.147 | |
|  | rs149805207 | | G | | A | | -0.072 | | 1.26E-02 | | 1.10E-08 | | -0.051 | | 0.071 | | 0.470 | | 0.00007 | | 32.685 | |
|  | rs17245213 | | A | | G | | -0.015 | | 2.61E-03 | | 2.00E-08 | | -0.017 | | 0.026 | | 0.526 | | 0.00007 | | 31.521 | |
|  | rs17576658 | | A | | G | | -0.013 | | 2.46E-03 | | 4.10E-08 | | -0.028 | | 0.024 | | 0.244 | | 0.00007 | | 30.116 | |
|  | rs17685 | | A | | G | | 0.023 | | 2.36E-03 | | 1.60E-22 | | 0.014 | | 0.020 | | 0.509 | | 0.00021 | | 95.364 | |
|  | rs2117137 | | G | | A | | 0.013 | | 2.16E-03 | | 1.70E-09 | | -0.003 | | 0.020 | | 0.875 | | 0.00008 | | 36.338 | |
|  | rs2273447 | | T | | A | | 0.017 | | 2.63E-03 | | 3.30E-11 | | -0.012 | | 0.029 | | 0.691 | | 0.00010 | | 43.990 | |
|  | rs2279844 | | A | | G | | -0.012 | | 2.18E-03 | | 4.00E-08 | | 0.024 | | 0.021 | | 0.243 | | 0.00007 | | 30.151 | |
|  | rs2351187 | | A | | G | | 0.013 | | 2.28E-03 | | 1.60E-08 | | 0.007 | | 0.021 | | 0.747 | | 0.00007 | | 31.959 | |
|  | rs2472297 | | T | | C | | 0.053 | | 2.40E-03 | | 2.30E-109 | | 0.004 | | 0.023 | | 0.871 | | 0.00110 | | 493.643 | |
|  | rs2478875 | | G | | A | | 0.022 | | 2.61E-03 | | 5.10E-17 | | 0.012 | | 0.027 | | 0.649 | | 0.00016 | | 70.299 | |
|  | rs2645929 | | G | | A | | -0.015 | | 2.72E-03 | | 3.50E-08 | | 0.007 | | 0.028 | | 0.803 | | 0.00007 | | 30.424 | |
|  | rs34619 | | A | | G | | 0.012 | | 2.14E-03 | | 4.30E-08 | | 0.041 | | 0.020 | | 0.044 | | 0.00007 | | 30.021 | |
|  | rs4410790 | | C | | T | | 0.041 | | 2.20E-03 | | 3.40E-76 | | 0.054 | | 0.021 | | 0.011 | | 0.00076 | | 341.268 | |
|  | rs4808193 | | C | | T | | 0.015 | | 2.25E-03 | | 1.70E-11 | | 0.036 | | 0.023 | | 0.120 | | 0.00010 | | 45.240 | |
|  | rs4817505 | | C | | T | | 0.015 | | 2.17E-03 | | 4.20E-12 | | -0.040 | | 0.020 | | 0.048 | | 0.00011 | | 48.012 | |
|  | rs56188862 | | C | | T | | -0.016 | | 2.17E-03 | | 4.30E-13 | | -0.015 | | 0.021 | | 0.480 | | 0.00012 | | 52.497 | |
|  | rs56348300 | | G | | C | | 0.016 | | 2.73E-03 | | 6.10E-09 | | -0.027 | | 0.030 | | 0.364 | | 0.00008 | | 33.799 | |
|  | rs57462170 | | A | | G | | 0.019 | | 3.41E-03 | | 1.90E-08 | | 0.031 | | 0.029 | | 0.284 | | 0.00007 | | 31.620 | |
|  | rs57631352 | | G | | A | | -0.013 | | 2.32E-03 | | 1.70E-08 | | -0.036 | | 0.021 | | 0.092 | | 0.00007 | | 31.868 | |
|  | rs6829 | | T | | C | | -0.012 | | 2.17E-03 | | 3.70E-08 | | -0.030 | | 0.020 | | 0.144 | | 0.00007 | | 30.282 | |
|  | rs713598 | | G | | C | | 0.013 | | 2.16E-03 | | 5.20E-10 | | -0.025 | | 0.021 | | 0.237 | | 0.00009 | | 38.590 | |
|  | rs72797284 | | G | | A | | -0.017 | | 2.38E-03 | | 7.00E-13 | | -0.028 | | 0.025 | | 0.251 | | 0.00012 | | 51.558 | |
|  | rs7757102 | | G | | A | | -0.012 | | 2.13E-03 | | 3.10E-08 | | -0.001 | | 0.020 | | 0.957 | | 0.00007 | | 30.624 | |
|  | rs9302428 | | G | | C | | 0.012 | | 2.20E-03 | | 2.60E-08 | | 0.017 | | 0.021 | | 0.426 | | 0.00007 | | 30.948 | |
|  | rs9624470 | | A | | G | | 0.025 | | 2.15E-03 | | 1.30E-31 | | 0.030 | | 0.020 | | 0.136 | | 0.00031 | | 136.839 | |
|  | rs9648476 | | A | | G | | 0.013 | | 2.19E-03 | | 1.10E-08 | | 0.012 | | 0.021 | | 0.578 | | 0.00007 | | 32.722 | |
|  | rs977474 | | T | | C | | 0.022 | | 2.86E-03 | | 2.40E-14 | | -0.008 | | 0.036 | | 0.823 | | 0.00013 | | 58.180 | |
| Poultry intake | rs2426440 | | G | | A | | 0.011 | | 2.05E-03 | | 4.70E-08 | | 0.010 | | 0.024 | | 0.684 | | 0.00006 | | 29.849 | |
|  | rs2565017 | | A | | G | | 0.011 | | 1.88E-03 | | 5.90E-09 | | 0.039 | | 0.021 | | 0.064 | | 0.00007 | | 33.858 | |
|  | rs2965200 | | A | | G | | -0.010 | | 1.90E-03 | | 4.20E-08 | | -0.031 | | 0.023 | | 0.175 | | 0.00007 | | 30.047 | |
|  | rs7829800 | | G | | A | | 0.011 | | 1.94E-03 | | 3.70E-09 | | -0.021 | | 0.023 | | 0.352 | | 0.00008 | | 34.781 | |
|  | rs9923768 | | A | | G | | 0.011 | | 1.86E-03 | | 1.60E-08 | | 0.024 | | 0.020 | | 0.240 | | 0.00007 | | 31.977 | |
|  | rs9997448 | | T | | C | | -0.010 | | 1.88E-03 | | 2.70E-08 | | -0.033 | | 0.021 | | 0.113 | | 0.00007 | | 30.889 | |
| Table1.2 SNPs used as instrumental variables from dietary factors and preeclampsia GWASs | | | | | | | | | | | | | | | | | | | | | | |
| **Traits** | | **SNPs** | | **Effect** | | **Other** | | **Dietary factors** | | | | | | **Preeclampsia** | | | | | | $\mathbf{R}^{\boldsymbol{2}}$ | | **F** |
|  |  |  |  | **allele** | | **allele** | | **Beta** | | **SE** | | ***P*-value** | | **Beta** | | **SE** | | ***P*-value** | |  |  |  |
| Cheese intake | | rs10896050 | | T | | G | | -0.018 | | 2.83E-03 | | 7.20E-11 | | -0.019 | | 0.034 | | 0.578 | | 0.00009 | | 42.461 |
|  | | rs113367286 | | T | | C | | 0.015 | | 2.50E-03 | | 1.30E-09 | | 0.002 | | 0.026 | | 0.950 | | 0.00008 | | 36.872 |
|  | | rs11620149 | | C | | T | | -0.018 | | 3.21E-03 | | 3.60E-08 | | 0.113 | | 0.040 | | 0.004 | | 0.00007 | | 30.345 |
|  | | rs11649653 | | G | | C | | 0.014 | | 2.29E-03 | | 1.50E-09 | | -0.014 | | 0.024 | | 0.554 | | 0.00008 | | 36.517 |
|  | | rs12296440 | | A | | G | | 0.019 | | 2.98E-03 | | 2.80E-10 | | 0.041 | | 0.028 | | 0.150 | | 0.00009 | | 39.779 |
|  | | rs12447542 | | A | | G | | 0.020 | | 3.41E-03 | | 6.80E-09 | | 0.000 | | 0.034 | | 0.999 | | 0.00007 | | 33.596 |
|  | | rs12672200 | | A | | G | | -0.014 | | 2.39E-03 | | 9.00E-09 | | -0.049 | | 0.025 | | 0.047 | | 0.00007 | | 33.037 |
|  | | rs12786959 | | T | | A | | -0.016 | | 2.82E-03 | | 1.20E-08 | | 0.035 | | 0.032 | | 0.273 | | 0.00007 | | 32.481 |
|  | | rs12951057 | | G | | C | | -0.021 | | 3.04E-03 | | 3.60E-12 | | 0.041 | | 0.028 | | 0.147 | | 0.00011 | | 48.309 |
|  | | rs1434511 | | T | | C | | 0.013 | | 2.26E-03 | | 9.50E-09 | | 0.000 | | 0.024 | | 0.999 | | 0.00007 | | 32.946 |
|  | | rs1514755 | | G | | A | | 0.016 | | 2.62E-03 | | 3.90E-10 | | 0.022 | | 0.026 | | 0.403 | | 0.00009 | | 39.166 |
|  | | rs17115145 | | T | | C | | -0.013 | | 2.29E-03 | | 1.80E-08 | | 0.008 | | 0.024 | | 0.757 | | 0.00007 | | 31.649 |
|  | | rs1806771 | | G | | T | | -0.022 | | 4.04E-03 | | 4.10E-08 | | 0.049 | | 0.045 | | 0.274 | | 0.00007 | | 30.125 |
|  | | rs1931805 | | C | | T | | 0.013 | | 2.24E-03 | | 1.60E-08 | | -0.010 | | 0.024 | | 0.688 | | 0.00007 | | 31.940 |
|  | | rs2339928 | | A | | G | | 0.015 | | 2.44E-03 | | 1.20E-09 | | -0.027 | | 0.028 | | 0.334 | | 0.00008 | | 36.929 |
|  | | rs2854175 | | A | | C | | 0.017 | | 2.57E-03 | | 3.70E-11 | | -0.073 | | 0.029 | | 0.010 | | 0.00010 | | 43.771 |
|  | | rs35270670 | | G | | A | | 0.016 | | 2.71E-03 | | 1.50E-09 | | 0.004 | | 0.029 | | 0.894 | | 0.00008 | | 36.537 |
|  | | rs3911016 | | G | | T | | 0.021 | | 3.44E-03 | | 5.30E-10 | | 0.036 | | 0.032 | | 0.265 | | 0.00009 | | 38.548 |
|  | | rs4296548 | | G | | T | | 0.013 | | 2.29E-03 | | 1.20E-08 | | -0.040 | | 0.024 | | 0.091 | | 0.00007 | | 32.410 |
|  | | rs4681981 | | A | | C | | -0.012 | | 2.24E-03 | | 2.90E-08 | | 0.014 | | 0.024 | | 0.556 | | 0.00007 | | 30.789 |
|  | | rs4692708 | | C | | A | | 0.015 | | 2.59E-03 | | 1.30E-08 | | -0.032 | | 0.030 | | 0.296 | | 0.00007 | | 32.368 |
|  | | rs4860341 | | C | | T | | 0.024 | | 4.35E-03 | | 2.20E-08 | | 0.039 | | 0.042 | | 0.347 | | 0.00007 | | 31.351 |
|  | | rs524468 | | G | | A | | -0.014 | | 2.55E-03 | | 2.40E-08 | | 0.020 | | 0.025 | | 0.438 | | 0.00007 | | 31.154 |
|  | | rs6126641 | | A | | G | | 0.013 | | 2.39E-03 | | 3.30E-08 | | 0.011 | | 0.024 | | 0.663 | | 0.00007 | | 30.498 |
|  | | rs61734410 | | T | | C | | 0.017 | | 2.62E-03 | | 2.20E-10 | | -0.036 | | 0.025 | | 0.156 | | 0.00009 | | 40.319 |
|  | | rs61953351 | | T | | G | | 0.015 | | 2.58E-03 | | 1.50E-08 | | 0.016 | | 0.026 | | 0.549 | | 0.00007 | | 31.994 |
|  | | rs62236533 | | A | | G | | 0.025 | | 3.65E-03 | | 1.10E-11 | | -0.039 | | 0.054 | | 0.475 | | 0.00010 | | 46.105 |
|  | | rs62245792 | | A | | T | | -0.018 | | 3.16E-03 | | 1.40E-08 | | 0.043 | | 0.028 | | 0.131 | | 0.00007 | | 32.145 |
|  | | rs6685323 | | T | | C | | -0.013 | | 2.42E-03 | | 4.80E-08 | | -0.031 | | 0.025 | | 0.216 | | 0.00007 | | 29.801 |
|  | | rs67238148 | | T | | G | | 0.017 | | 2.71E-03 | | 1.10E-09 | | 0.009 | | 0.029 | | 0.762 | | 0.00008 | | 37.146 |
|  | | rs6774906 | | C | | A | | 0.032 | | 5.67E-03 | | 2.50E-08 | | -0.032 | | 0.059 | | 0.581 | | 0.00007 | | 31.089 |
|  | | rs6873324 | | C | | A | | -0.012 | | 2.27E-03 | | 3.90E-08 | | -0.009 | | 0.024 | | 0.726 | | 0.00007 | | 30.190 |
|  | | rs7012814 | | A | | G | | -0.019 | | 2.25E-03 | | 2.10E-16 | | 0.016 | | 0.027 | | 0.552 | | 0.00015 | | 67.489 |
|  | | rs71386942 | | A | | C | | 0.014 | | 2.52E-03 | | 9.90E-09 | | -0.044 | | 0.028 | | 0.114 | | 0.00007 | | 32.851 |
|  | | rs72810360 | | T | | C | | 0.017 | | 2.95E-03 | | 1.30E-08 | | -0.040 | | 0.030 | | 0.180 | | 0.00007 | | 32.359 |
|  | | rs72970243 | | A | | G | | 0.022 | | 3.40E-03 | | 6.70E-11 | | -0.018 | | 0.032 | | 0.569 | | 0.00009 | | 42.596 |
|  | | rs7298331 | | C | | A | | -0.013 | | 2.31E-03 | | 1.10E-08 | | 0.020 | | 0.024 | | 0.400 | | 0.00007 | | 32.646 |
|  | | rs73024305 | | C | | G | | 0.033 | | 4.93E-03 | | 4.00E-11 | | -0.038 | | 0.063 | | 0.544 | | 0.00010 | | 43.608 |
|  | | rs73096946 | | C | | T | | -0.021 | | 3.07E-03 | | 1.90E-11 | | 0.041 | | 0.042 | | 0.324 | | 0.00010 | | 45.086 |
|  | | rs73335955 | | C | | T | | 0.028 | | 4.98E-03 | | 2.40E-08 | | -0.084 | | 0.062 | | 0.175 | | 0.00007 | | 31.123 |
|  | | rs7386207 | | T | | C | | -0.012 | | 2.27E-03 | | 3.60E-08 | | -0.018 | | 0.025 | | 0.470 | | 0.00007 | | 30.330 |
|  | | rs77742462 | | G | | A | | -0.047 | | 8.28E-03 | | 9.80E-09 | | 0.015 | | 0.084 | | 0.862 | | 0.00007 | | 32.879 |
|  | | rs78876700 | | A | | G | | 0.018 | | 3.28E-03 | | 3.40E-08 | | -0.021 | | 0.043 | | 0.628 | | 0.00007 | | 30.467 |
|  | | rs79184944 | | A | | T | | 0.020 | | 3.28E-03 | | 2.40E-09 | | -0.001 | | 0.028 | | 0.969 | | 0.00008 | | 35.615 |
|  | | rs7936836 | | A | | C | | 0.016 | | 2.27E-03 | | 2.60E-12 | | -0.008 | | 0.024 | | 0.749 | | 0.00011 | | 48.962 |
|  | | rs919109 | | C | | G | | 0.020 | | 3.24E-03 | | 7.90E-10 | | 0.013 | | 0.032 | | 0.692 | | 0.00008 | | 37.792 |
|  | | rs9504123 | | C | | A | | 0.014 | | 2.50E-03 | | 1.50E-08 | | 0.016 | | 0.026 | | 0.545 | | 0.00007 | | 32.030 |
| Lamb/mutton intake | | rs11090045 | | A | | G | | -0.011 | | 1.61E-03 | | 3.00E-11 | | 0.016 | | 0.025 | | 0.513 | | 0.00010 | | 44.159 |
|  | | rs12634740 | | G | | T | | -0.010 | | 1.70E-03 | | 2.80E-09 | | 0.044 | | 0.026 | | 0.087 | | 0.00008 | | 35.324 |
|  | | rs136548 | | T | | C | | 0.010 | | 1.51E-03 | | 2.90E-10 | | -0.003 | | 0.024 | | 0.915 | | 0.00009 | | 39.732 |
|  | | rs139237013 | | A | | G | | 0.019 | | 3.14E-03 | | 1.80E-09 | | -0.002 | | 0.059 | | 0.974 | | 0.00008 | | 36.224 |
|  | | rs1556147 | | T | | A | | 0.009 | | 1.56E-03 | | 5.30E-09 | | 0.080 | | 0.028 | | 0.004 | | 0.00007 | | 34.078 |
|  | | rs16891982 | | G | | C | | -0.024 | | 4.36E-03 | | 2.70E-08 | | 0.000 | | 0.091 | | 0.997 | | 0.00007 | | 30.919 |
|  | | rs17270057 | | C | | T | | 0.013 | | 2.31E-03 | | 4.30E-08 | | 0.010 | | 0.048 | | 0.830 | | 0.00007 | | 30.031 |
|  | | rs1958801 | | G | | A | | -0.009 | | 1.62E-03 | | 3.20E-08 | | 0.014 | | 0.028 | | 0.616 | | 0.00007 | | 30.575 |
|  | | rs2222760 | | A | | G | | -0.009 | | 1.64E-03 | | 2.80E-08 | | 0.023 | | 0.028 | | 0.410 | | 0.00007 | | 30.844 |
|  | | rs2678900 | | G | | T | | 0.010 | | 1.48E-03 | | 9.90E-12 | | 0.030 | | 0.024 | | 0.216 | | 0.00010 | | 46.353 |
|  | | rs2726033 | | G | | A | | -0.009 | | 1.48E-03 | | 1.50E-10 | | 0.049 | | 0.024 | | 0.040 | | 0.00009 | | 41.090 |
|  | | rs276453 | | C | | A | | -0.014 | | 1.47E-03 | | 2.90E-22 | | 0.008 | | 0.024 | | 0.748 | | 0.00020 | | 94.154 |
|  | | rs2926119 | | A | | C | | 0.008 | | 1.48E-03 | | 4.40E-08 | | 0.035 | | 0.024 | | 0.141 | | 0.00007 | | 29.964 |
|  | | rs3105056 | | C | | T | | -0.012 | | 1.65E-03 | | 1.80E-12 | | 0.040 | | 0.028 | | 0.155 | | 0.00011 | | 49.718 |
|  | | rs35797675 | | G | | T | | -0.011 | | 1.79E-03 | | 1.40E-09 | | 0.000 | | 0.031 | | 0.989 | | 0.00008 | | 36.601 |
|  | | rs3909726 | | A | | G | | 0.014 | | 1.98E-03 | | 1.80E-12 | | -0.053 | | 0.032 | | 0.099 | | 0.00011 | | 49.715 |
|  | | rs3964074 | | C | | T | | -0.008 | | 1.47E-03 | | 3.20E-08 | | 0.030 | | 0.024 | | 0.200 | | 0.00007 | | 30.581 |
|  | | rs4272399 | | A | | C | | -0.009 | | 1.58E-03 | | 4.50E-09 | | -0.038 | | 0.025 | | 0.118 | | 0.00007 | | 34.406 |
|  | | rs429358 | | C | | T | | -0.018 | | 2.03E-03 | | 2.70E-19 | | 0.056 | | 0.031 | | 0.068 | | 0.00018 | | 80.611 |
|  | | rs55813438 | | A | | G | | -0.011 | | 1.74E-03 | | 4.70E-11 | | -0.014 | | 0.029 | | 0.623 | | 0.00009 | | 43.300 |
|  | | rs56394517 | | G | | A | | -0.014 | | 2.49E-03 | | 3.20E-08 | | 0.016 | | 0.041 | | 0.696 | | 0.00007 | | 30.573 |
|  | | rs62106258 | | C | | T | | 0.022 | | 3.40E-03 | | 2.00E-10 | | -0.137 | | 0.085 | | 0.107 | | 0.00009 | | 40.437 |
|  | | rs62398404 | | T | | C | | 0.013 | | 2.19E-03 | | 4.00E-09 | | -0.006 | | 0.035 | | 0.855 | | 0.00008 | | 34.642 |
|  | | rs6581296 | | G | | C | | 0.010 | | 1.82E-03 | | 4.00E-08 | | 0.015 | | 0.028 | | 0.594 | | 0.00007 | | 30.166 |
|  | | rs660880 | | A | | G | | -0.009 | | 1.46E-03 | | 6.80E-10 | | 0.037 | | 0.024 | | 0.116 | | 0.00008 | | 38.064 |
|  | | rs673696 | | T | | C | | 0.016 | | 2.68E-03 | | 3.70E-09 | | -0.041 | | 0.044 | | 0.345 | | 0.00008 | | 34.796 |
|  | | rs6829572 | | A | | G | | 0.008 | | 1.47E-03 | | 1.20E-08 | | -0.007 | | 0.025 | | 0.765 | | 0.00007 | | 32.481 |
|  | | rs7447465 | | C | | T | | 0.010 | | 1.51E-03 | | 2.00E-10 | | -0.001 | | 0.025 | | 0.985 | | 0.00009 | | 40.474 |
|  | | rs7550173 | | T | | A | | -0.009 | | 1.50E-03 | | 1.30E-09 | | -0.002 | | 0.025 | | 0.948 | | 0.00008 | | 36.884 |
|  | | rs994270 | | G | | C | | 0.010 | | 1.73E-03 | | 1.40E-08 | | -0.037 | | 0.031 | | 0.225 | | 0.00007 | | 32.242 |

Table1.3 SNPs used as instrumental variables from dietary factors and gestational diabetes mellitus GWASs

| **Traits** | **SNPs** | **Effect** | **Other** | **Dietary factors** | | | **Gestational diabetes mellitus** | | | $\mathbf{R}^{\boldsymbol{2}}$ | **F** |
| --- | --- | --- | --- | --- | --- | --- | --- | --- | --- | --- | --- |
|  |  | **allele** | **allele** | **Beta** | **SE** | ***P*-value** | **Beta** | **SE** | ***P*-value** |  |  |
| Cheese intake | rs1073242 | A | G | 0.016 | 2.29E-03 | 6.70E-12 | -0.051 | 0.021 | 0.012 | 0.00010 | 47.107 |
|  | rs10896050 | T | G | -0.018 | 2.83E-03 | 7.20E-11 | 0.024 | 0.029 | 0.403 | 0.00009 | 42.461 |
|  | rs113367286 | T | C | 0.015 | 2.50E-03 | 1.30E-09 | -0.039 | 0.022 | 0.074 | 0.00008 | 36.872 |
|  | rs11620149 | C | T | -0.018 | 3.21E-03 | 3.60E-08 | 0.086 | 0.034 | 0.010 | 0.00007 | 30.345 |
|  | rs11649653 | G | C | 0.014 | 2.29E-03 | 1.50E-09 | -0.029 | 0.021 | 0.161 | 0.00008 | 36.517 |
|  | rs12296440 | A | G | 0.019 | 2.98E-03 | 2.80E-10 | -0.013 | 0.024 | 0.582 | 0.00009 | 39.779 |
|  | rs12447542 | A | G | 0.020 | 3.41E-03 | 6.80E-09 | 0.033 | 0.029 | 0.257 | 0.00007 | 33.596 |
|  | rs12672200 | A | G | -0.014 | 2.39E-03 | 9.00E-09 | -0.035 | 0.021 | 0.101 | 0.00007 | 33.037 |
|  | rs12786959 | T | A | -0.016 | 2.82E-03 | 1.20E-08 | 0.031 | 0.027 | 0.261 | 0.00007 | 32.481 |
|  | rs1291145 | C | T | -0.020 | 2.41E-03 | 4.40E-17 | 0.036 | 0.021 | 0.080 | 0.00016 | 70.583 |
|  | rs12951057 | G | C | -0.021 | 3.04E-03 | 3.60E-12 | 0.019 | 0.024 | 0.426 | 0.00011 | 48.309 |
|  | rs1434511 | T | C | 0.013 | 2.26E-03 | 9.50E-09 | 0.001 | 0.020 | 0.955 | 0.00007 | 32.946 |
|  | rs1514755 | G | A | 0.016 | 2.62E-03 | 3.90E-10 | -0.038 | 0.022 | 0.089 | 0.00009 | 39.166 |
|  | rs17115145 | T | C | -0.013 | 2.29E-03 | 1.80E-08 | -0.007 | 0.021 | 0.739 | 0.00007 | 31.649 |
|  | rs1806771 | G | T | -0.022 | 4.04E-03 | 4.10E-08 | 0.077 | 0.038 | 0.041 | 0.00007 | 30.125 |
|  | rs1931805 | C | T | 0.013 | 2.24E-03 | 1.60E-08 | -0.008 | 0.020 | 0.681 | 0.00007 | 31.940 |
|  | rs2339928 | A | G | 0.015 | 2.44E-03 | 1.20E-09 | -0.025 | 0.024 | 0.279 | 0.00008 | 36.929 |
|  | rs26579 | C | G | -0.013 | 2.29E-03 | 2.40E-08 | 0.019 | 0.021 | 0.365 | 0.00007 | 31.130 |
|  | rs2802530 | A | G | 0.019 | 3.40E-03 | 4.20E-08 | -0.070 | 0.028 | 0.012 | 0.00007 | 30.065 |
|  | rs2854175 | A | C | 0.017 | 2.57E-03 | 3.70E-11 | -0.016 | 0.024 | 0.507 | 0.00010 | 43.771 |
|  | rs2960578 | G | T | 0.017 | 2.24E-03 | 2.60E-14 | -0.071 | 0.020 | 0.000 | 0.00013 | 57.998 |
|  | rs34198643 | T | C | -0.017 | 2.68E-03 | 4.50E-10 | -0.025 | 0.028 | 0.364 | 0.00009 | 38.874 |
|  | rs35270670 | G | A | 0.016 | 2.71E-03 | 1.50E-09 | 0.015 | 0.024 | 0.526 | 0.00008 | 36.537 |
|  | rs3911016 | G | T | 0.021 | 3.44E-03 | 5.30E-10 | -0.016 | 0.028 | 0.557 | 0.00009 | 38.548 |
|  | rs4296548 | G | T | 0.013 | 2.29E-03 | 1.20E-08 | -0.007 | 0.020 | 0.717 | 0.00007 | 32.410 |
|  | rs4503172 | T | C | 0.013 | 2.29E-03 | 1.60E-08 | -0.028 | 0.021 | 0.170 | 0.00007 | 31.943 |
|  | rs4681981 | A | C | -0.012 | 2.24E-03 | 2.90E-08 | 0.013 | 0.020 | 0.528 | 0.00007 | 30.789 |
|  | rs4692708 | C | A | 0.015 | 2.59E-03 | 1.30E-08 | 0.024 | 0.026 | 0.349 | 0.00007 | 32.368 |
|  | rs4860341 | C | T | 0.024 | 4.35E-03 | 2.20E-08 | -0.052 | 0.036 | 0.144 | 0.00007 | 31.351 |
|  | rs504675 | T | C | 0.027 | 2.34E-03 | 1.00E-31 | 0.003 | 0.020 | 0.881 | 0.00030 | 137.314 |
|  | rs524468 | G | A | -0.014 | 2.55E-03 | 2.40E-08 | 0.019 | 0.022 | 0.380 | 0.00007 | 31.154 |
|  | rs531358 | T | C | 0.013 | 2.34E-03 | 1.80E-08 | 0.008 | 0.021 | 0.700 | 0.00007 | 31.733 |
|  | rs6126641 | A | G | 0.013 | 2.39E-03 | 3.30E-08 | -0.060 | 0.021 | 0.004 | 0.00007 | 30.498 |
|  | rs61734410 | T | C | 0.017 | 2.62E-03 | 2.20E-10 | -0.010 | 0.022 | 0.645 | 0.00009 | 40.319 |
|  | rs61953351 | T | G | 0.015 | 2.58E-03 | 1.50E-08 | -0.018 | 0.022 | 0.413 | 0.00007 | 31.994 |
|  | rs62034322 | A | G | -0.014 | 2.30E-03 | 1.40E-09 | -0.025 | 0.020 | 0.215 | 0.00008 | 36.715 |
|  | rs62236533 | A | G | 0.025 | 3.65E-03 | 1.10E-11 | -0.036 | 0.046 | 0.427 | 0.00010 | 46.105 |
|  | rs62245792 | A | T | -0.018 | 3.16E-03 | 1.40E-08 | 0.035 | 0.024 | 0.150 | 0.00007 | 32.145 |
|  | rs6685323 | T | C | -0.013 | 2.42E-03 | 4.80E-08 | 0.017 | 0.021 | 0.418 | 0.00007 | 29.801 |
|  | rs67238148 | T | G | 0.017 | 2.71E-03 | 1.10E-09 | -0.023 | 0.025 | 0.361 | 0.00008 | 37.146 |
|  | rs6774906 | C | A | 0.032 | 5.67E-03 | 2.50E-08 | -0.066 | 0.050 | 0.187 | 0.00007 | 31.089 |
|  | rs6873324 | C | A | -0.012 | 2.27E-03 | 3.90E-08 | 0.000 | 0.021 | 0.986 | 0.00007 | 30.190 |
|  | rs7012814 | A | G | -0.019 | 2.25E-03 | 2.10E-16 | -0.037 | 0.023 | 0.107 | 0.00015 | 67.489 |
|  | rs71386942 | A | C | 0.014 | 2.52E-03 | 9.90E-09 | 0.003 | 0.024 | 0.904 | 0.00007 | 32.851 |
|  | rs72810360 | T | C | 0.017 | 2.95E-03 | 1.30E-08 | 0.004 | 0.026 | 0.889 | 0.00007 | 32.359 |
|  | rs72970243 | A | G | 0.022 | 3.40E-03 | 6.70E-11 | -0.032 | 0.027 | 0.230 | 0.00009 | 42.596 |
|  | rs7298331 | C | A | -0.013 | 2.31E-03 | 1.10E-08 | -0.012 | 0.020 | 0.569 | 0.00007 | 32.646 |
|  | rs73024305 | C | G | 0.033 | 4.93E-03 | 4.00E-11 | 0.010 | 0.054 | 0.849 | 0.00010 | 43.608 |
|  | rs73096946 | C | T | -0.021 | 3.07E-03 | 1.90E-11 | 0.084 | 0.035 | 0.018 | 0.00010 | 45.086 |
|  | rs73335955 | C | T | 0.028 | 4.98E-03 | 2.40E-08 | -0.057 | 0.052 | 0.273 | 0.00007 | 31.123 |
|  | rs7386207 | T | C | -0.012 | 2.27E-03 | 3.60E-08 | -0.032 | 0.022 | 0.132 | 0.00007 | 30.330 |
|  | rs77742462 | G | A | -0.047 | 8.28E-03 | 9.80E-09 | 0.050 | 0.071 | 0.479 | 0.00007 | 32.879 |
|  | rs78876700 | A | G | 0.018 | 3.28E-03 | 3.40E-08 | 0.038 | 0.036 | 0.299 | 0.00007 | 30.467 |
|  | rs79184944 | A | T | 0.020 | 3.28E-03 | 2.40E-09 | -0.011 | 0.024 | 0.642 | 0.00008 | 35.615 |
|  | rs7936836 | A | C | 0.016 | 2.27E-03 | 2.60E-12 | -0.062 | 0.020 | 0.002 | 0.00011 | 48.962 |
|  | rs919109 | C | G | 0.020 | 3.24E-03 | 7.90E-10 | 0.028 | 0.027 | 0.303 | 0.00008 | 37.792 |
|  | rs9504123 | C | A | 0.014 | 2.50E-03 | 1.50E-08 | -0.007 | 0.022 | 0.767 | 0.00007 | 32.030 |
|  | rs975303 | G | A | 0.021 | 2.91E-03 | 2.50E-13 | -0.030 | 0.028 | 0.284 | 0.00012 | 53.564 |
|  | | | | | | | | | | | |

Table1.4 SNPs used as instrumental variables from dietary factors and fetal growth restriction GWASs

| **Traits** | **SNPs** | **Effect** | **Other** | **Dietary factors** | | | **Fetal growth restriction** | | | **R2** | **F** |
| --- | --- | --- | --- | --- | --- | --- | --- | --- | --- | --- | --- |
|  |  | **allele** | **allele** | **Beta** | **SE** | ***P*-value** | **Beta** | **SE** | ***P*-value** |  |  |
| Oily fish intake | rs10061973 | T | G | -0.011 | 1.92E-03 | 1.50E-08 | 0.011 | 0.024 | 0.654 | 0.00007 | 32.081 |
|  | rs10076975 | C | T | 0.011 | 1.97E-03 | 1.10E-08 | -0.040 | 0.024 | 0.099 | 0.00007 | 32.643 |
|  | rs10510554 | C | T | 0.011 | 1.94E-03 | 1.20E-08 | 0.080 | 0.024 | 0.001 | 0.00007 | 32.533 |
|  | rs10513136 | A | G | -0.023 | 3.86E-03 | 1.60E-09 | -0.152 | 0.097 | 0.116 | 0.00008 | 36.451 |
|  | rs10828250 | G | C | -0.020 | 2.07E-03 | 2.60E-22 | 0.009 | 0.026 | 0.734 | 0.00020 | 94.409 |
|  | rs114497213 | T | G | 0.027 | 4.24E-03 | 1.10E-10 | 0.095 | 0.064 | 0.137 | 0.00009 | 41.602 |
|  | rs11607886 | C | T | -0.012 | 1.94E-03 | 2.90E-09 | -0.014 | 0.025 | 0.570 | 0.00008 | 35.221 |
|  | rs11767283 | G | A | 0.018 | 2.32E-03 | 2.50E-14 | 0.034 | 0.027 | 0.214 | 0.00013 | 58.060 |
|  | rs11859365 | C | A | 0.023 | 2.20E-03 | 9.40E-25 | -0.006 | 0.026 | 0.832 | 0.00023 | 105.527 |
|  | rs1201289 | G | T | -0.011 | 1.96E-03 | 4.40E-08 | 0.012 | 0.026 | 0.657 | 0.00007 | 29.966 |
|  | rs12663865 | A | G | 0.013 | 2.23E-03 | 1.10E-08 | 0.057 | 0.029 | 0.047 | 0.00007 | 32.732 |
|  | rs12896749 | C | G | -0.011 | 1.97E-03 | 2.50E-08 | -0.002 | 0.025 | 0.943 | 0.00007 | 31.099 |
|  | rs12983532 | T | C | -0.013 | 2.23E-03 | 2.00E-09 | -0.017 | 0.029 | 0.564 | 0.00008 | 35.944 |
|  | rs13070166 | A | T | 0.014 | 2.28E-03 | 4.40E-10 | 0.003 | 0.029 | 0.927 | 0.00008 | 38.911 |
|  | rs1361016 | G | T | 0.015 | 2.65E-03 | 1.70E-08 | -0.015 | 0.035 | 0.676 | 0.00007 | 31.784 |
|  | rs16891727 | A | C | -0.024 | 2.84E-03 | 6.80E-17 | -0.010 | 0.044 | 0.814 | 0.00015 | 69.740 |
|  | rs17050031 | T | C | -0.012 | 1.92E-03 | 3.50E-10 | 0.002 | 0.024 | 0.929 | 0.00009 | 39.355 |
|  | rs1876245 | C | T | 0.015 | 1.93E-03 | 5.00E-15 | 0.013 | 0.025 | 0.600 | 0.00013 | 61.266 |
|  | rs1951286 | G | T | -0.015 | 2.00E-03 | 3.00E-13 | -0.018 | 0.025 | 0.474 | 0.00012 | 53.196 |
|  | rs2271308 | C | T | 0.012 | 2.16E-03 | 3.10E-08 | 0.063 | 0.027 | 0.021 | 0.00007 | 30.630 |
|  | rs275160 | C | T | 0.012 | 2.10E-03 | 8.00E-09 | 0.033 | 0.026 | 0.204 | 0.00007 | 33.272 |
|  | rs2827161 | G | T | 0.011 | 1.94E-03 | 3.20E-08 | 0.030 | 0.025 | 0.245 | 0.00007 | 30.574 |
|  | rs28533540 | A | G | 0.015 | 1.92E-03 | 2.80E-14 | 0.028 | 0.024 | 0.259 | 0.00013 | 57.869 |
|  | rs28623270 | T | A | -0.018 | 2.74E-03 | 7.30E-11 | -0.004 | 0.038 | 0.916 | 0.00009 | 42.437 |
|  | rs303817 | G | A | 0.014 | 2.21E-03 | 8.00E-10 | -0.001 | 0.027 | 0.974 | 0.00008 | 37.770 |
|  | rs3124402 | G | A | -0.022 | 2.16E-03 | 1.90E-24 | -0.060 | 0.029 | 0.036 | 0.00023 | 104.087 |
|  | rs35287743 | T | G | -0.028 | 3.01E-03 | 7.00E-21 | -0.006 | 0.042 | 0.891 | 0.00019 | 87.857 |
|  | rs4002471 | T | C | -0.019 | 1.92E-03 | 1.50E-23 | -0.024 | 0.024 | 0.332 | 0.00022 | 99.999 |
|  | rs45501495 | T | C | 0.016 | 2.26E-03 | 3.70E-12 | 0.013 | 0.027 | 0.644 | 0.00010 | 48.261 |
|  | rs4869859 | C | T | 0.014 | 1.92E-03 | 3.10E-13 | -0.010 | 0.024 | 0.667 | 0.00012 | 53.128 |
|  | rs4982738 | A | G | 0.011 | 1.97E-03 | 3.50E-08 | 0.003 | 0.025 | 0.891 | 0.00007 | 30.414 |
|  | rs510161 | G | C | -0.011 | 2.07E-03 | 4.50E-08 | 0.005 | 0.029 | 0.862 | 0.00006 | 29.910 |
|  | rs552234 | A | G | -0.012 | 1.91E-03 | 1.10E-09 | -0.050 | 0.024 | 0.040 | 0.00008 | 37.089 |
|  | rs55930451 | T | C | -0.017 | 3.08E-03 | 2.90E-08 | -0.016 | 0.045 | 0.725 | 0.00007 | 30.744 |
|  | rs55985303 | A | G | 0.013 | 2.24E-03 | 6.60E-09 | 0.004 | 0.027 | 0.883 | 0.00007 | 33.657 |
|  | rs59355765 | T | C | -0.016 | 2.61E-03 | 4.70E-10 | -0.067 | 0.037 | 0.074 | 0.00008 | 38.784 |
|  | rs6033437 | A | C | 0.012 | 2.21E-03 | 1.70E-08 | 0.005 | 0.030 | 0.877 | 0.00007 | 31.836 |
|  | rs6059844 | G | A | 0.011 | 1.91E-03 | 9.20E-09 | -0.019 | 0.024 | 0.442 | 0.00007 | 33.012 |
|  | rs6089753 | T | C | -0.012 | 1.92E-03 | 1.80E-09 | 0.004 | 0.024 | 0.875 | 0.00008 | 36.198 |
|  | rs61882686 | A | C | 0.020 | 3.43E-03 | 8.00E-09 | -0.057 | 0.045 | 0.208 | 0.00007 | 33.264 |
|  | rs631490 | C | G | -0.015 | 2.10E-03 | 6.00E-13 | -0.055 | 0.028 | 0.054 | 0.00011 | 51.851 |
|  | rs6465487 | G | A | -0.012 | 1.96E-03 | 2.70E-10 | 0.022 | 0.024 | 0.376 | 0.00009 | 39.876 |
|  | rs703987 | C | G | 0.011 | 1.97E-03 | 1.70E-08 | 0.002 | 0.026 | 0.934 | 0.00007 | 31.756 |
|  | rs7225002 | G | A | -0.014 | 1.94E-03 | 8.10E-13 | 0.004 | 0.025 | 0.860 | 0.00011 | 51.253 |
|  | rs7243428 | G | A | -0.013 | 2.29E-03 | 1.50E-08 | 0.016 | 0.030 | 0.585 | 0.00007 | 32.060 |
|  | rs7254235 | G | A | -0.011 | 1.94E-03 | 4.30E-08 | -0.025 | 0.026 | 0.349 | 0.00007 | 30.022 |
|  | rs75887709 | G | A | -0.016 | 2.81E-03 | 1.60E-08 | 0.004 | 0.041 | 0.920 | 0.00007 | 31.950 |
|  | rs7683782 | G | C | 0.014 | 2.57E-03 | 1.90E-08 | -0.018 | 0.035 | 0.601 | 0.00007 | 31.635 |
|  | rs790564 | C | A | 0.015 | 2.15E-03 | 7.90E-12 | -0.030 | 0.028 | 0.284 | 0.00010 | 46.789 |
|  | rs905575 | G | C | 0.014 | 2.52E-03 | 3.60E-08 | 0.000 | 0.042 | 0.994 | 0.00007 | 30.368 |
|  | rs9301837 | A | C | -0.016 | 2.73E-03 | 8.10E-09 | 0.039 | 0.028 | 0.166 | 0.00007 | 33.242 |
|  | rs9597870 | G | T | -0.013 | 2.23E-03 | 1.10E-08 | 0.003 | 0.029 | 0.922 | 0.00007 | 32.650 |
|  | rs9606833 | C | T | 0.017 | 2.23E-03 | 2.70E-14 | 0.060 | 0.028 | 0.033 | 0.00013 | 57.971 |
|  | rs9841174 | C | T | 0.015 | 1.98E-03 | 8.50E-14 | -0.012 | 0.024 | 0.630 | 0.00012 | 55.692 |
|  | rs9889161 | T | G | -0.013 | 2.00E-03 | 2.80E-11 | -0.013 | 0.027 | 0.636 | 0.00010 | 44.290 |
|  | rs9958909 | G | T | 0.016 | 2.78E-03 | 1.40E-08 | -0.013 | 0.032 | 0.679 | 0.00007 | 32.189 |

Note: SE, standard error; SNP, single nucleotide polymorphism.
