# Supplementary material for: Dietary factors and risk for adverse pregnancy outcome: A Mendelian randomization analysis
Source: Food Sci Nutr. 2024 Aug 22;12(10):8150–8. doi: 10.1002/fsn3.4412 (PMC11521750; doi:10.1002/fsn3.4412)
Supplement: Supplementary file 1 — Appendix S1. [file FSN3-12-8150-s001.zip › fsn34412-sup-0007-Captions.docx]

**Figure and table captions：**

**Figure S1** Leave-one-out analysis of dietary factors on preterm birth

**Figure S2** Leave-one-out analysis of dietary factors on other adverse pregnancy outcomes

**Figure S3** Mendelian randomization analysis scatter plot of dietary factors on preterm birth

**Figure S4** Mendelian randomization analysis scatter plot of dietary factors on other adverse pregnancy outcomes

**Table S1** Dataset description
