# Supplementary figures and images for: Dietary factors and risk for adverse pregnancy outcome: A Mendelian randomization analysis
Source: Food Sci Nutr. 2024 Aug 22;12(10):8150–8. doi: 10.1002/fsn3.4412 (PMC11521750; doi:10.1002/fsn3.4412)

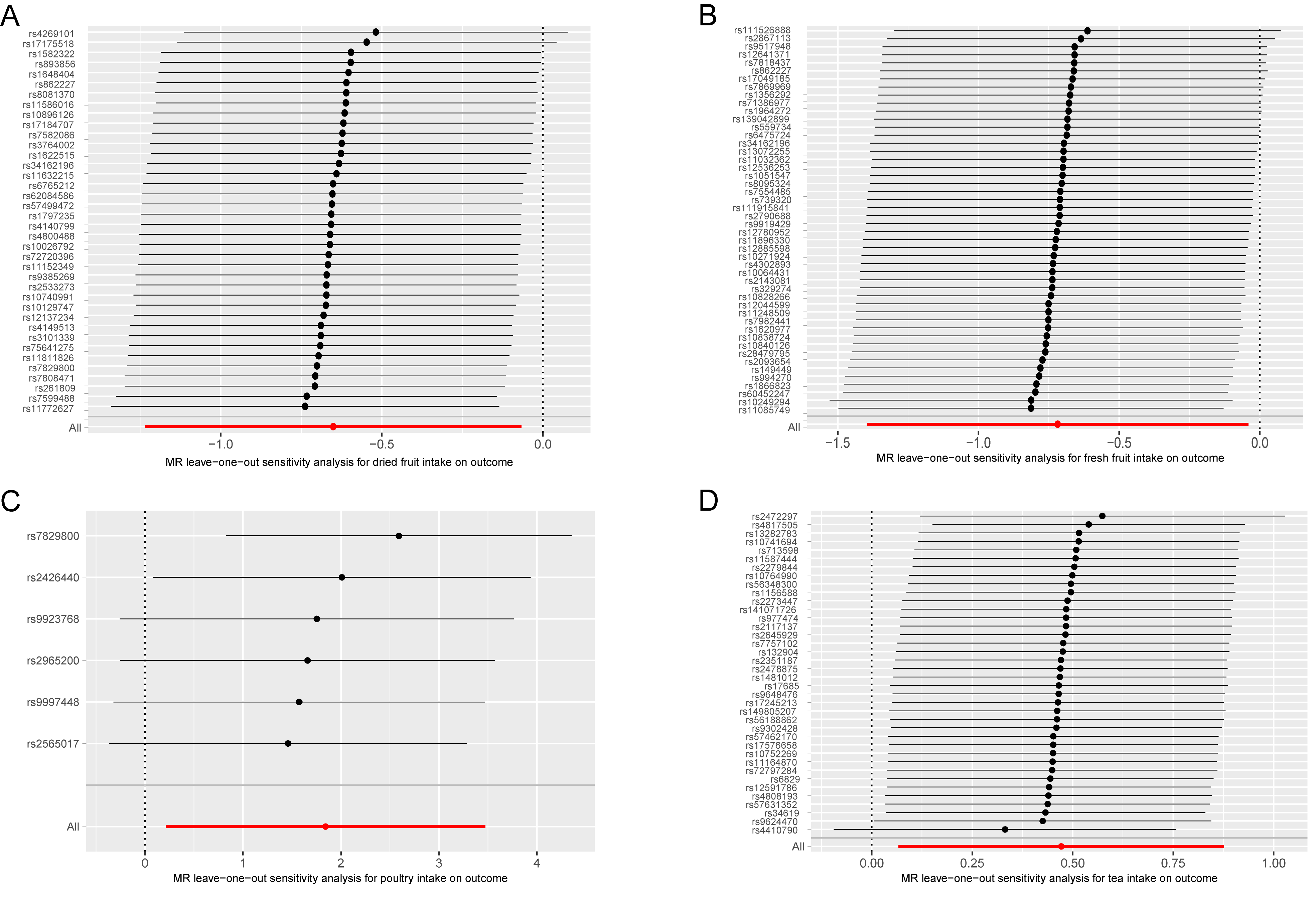

Supplement: Supplementary file 1 — Appendix S1. [file FSN3-12-8150-s001.zip › fsn34412-sup-0001-FigureS1.tif]

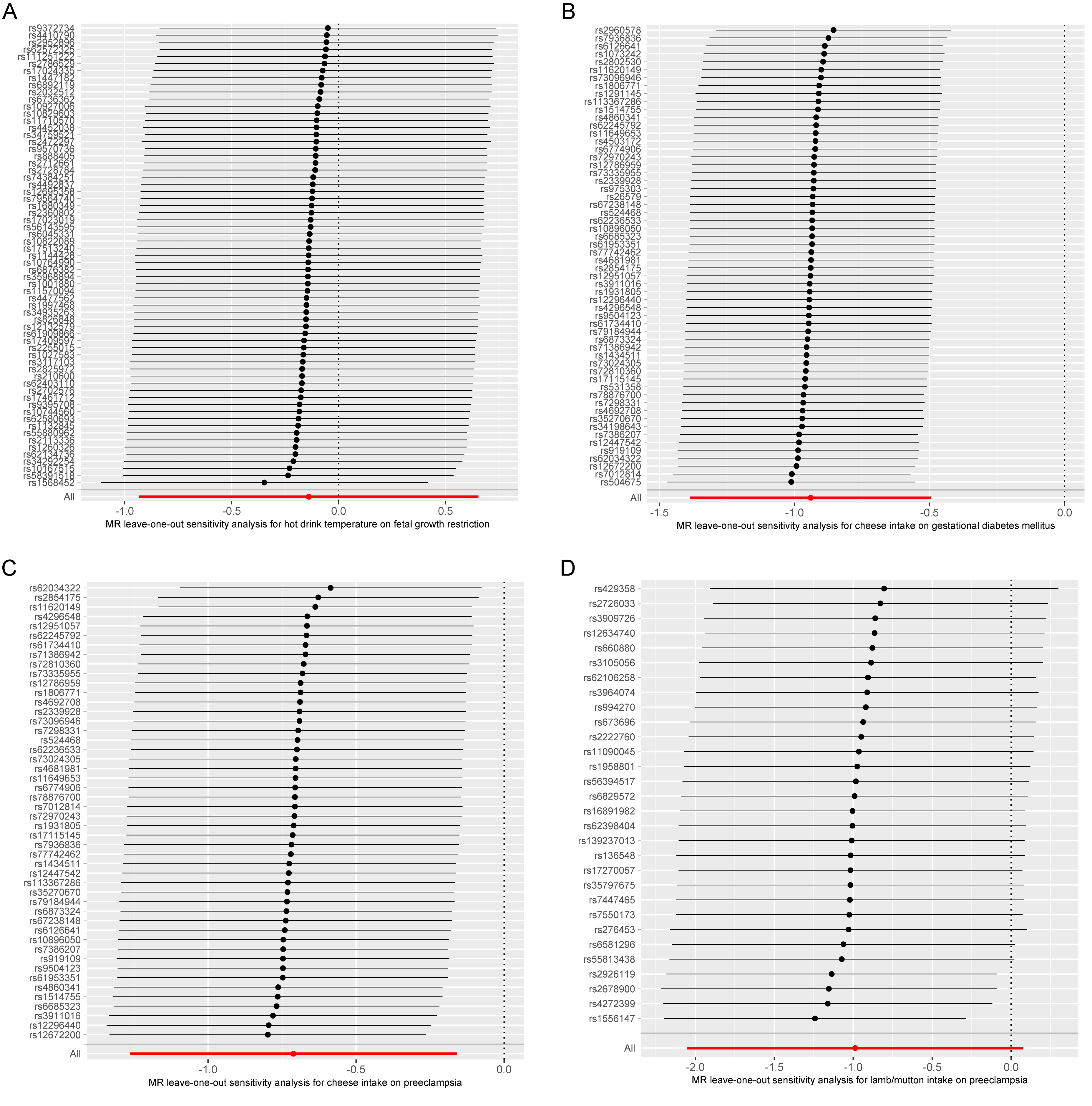

Supplement: Supplementary file 1 — Appendix S1. [file FSN3-12-8150-s001.zip › fsn34412-sup-0002-FigureS2.tif]

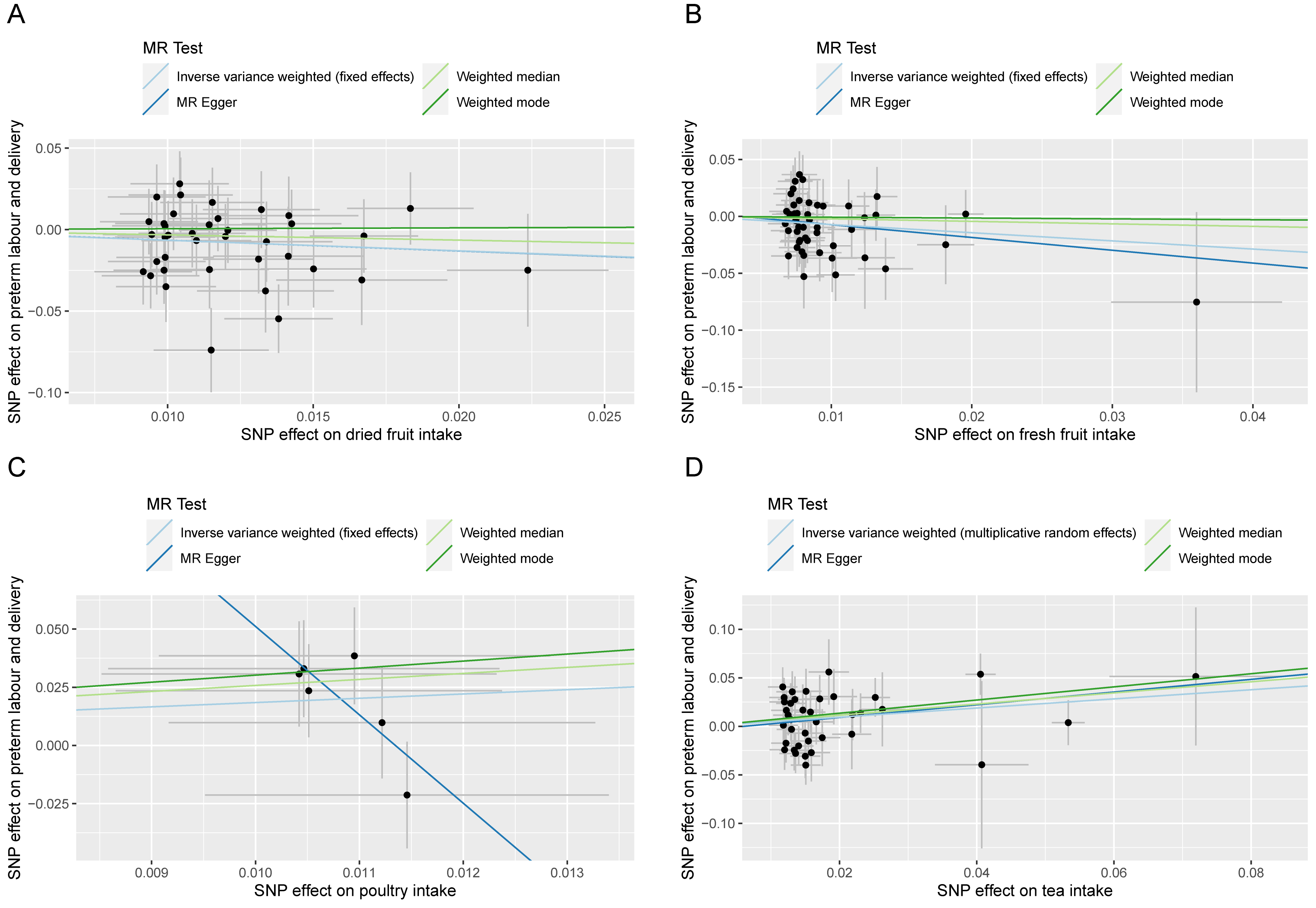

Supplement: Supplementary file 1 — Appendix S1. [file FSN3-12-8150-s001.zip › fsn34412-sup-0003-FigureS3.tif]

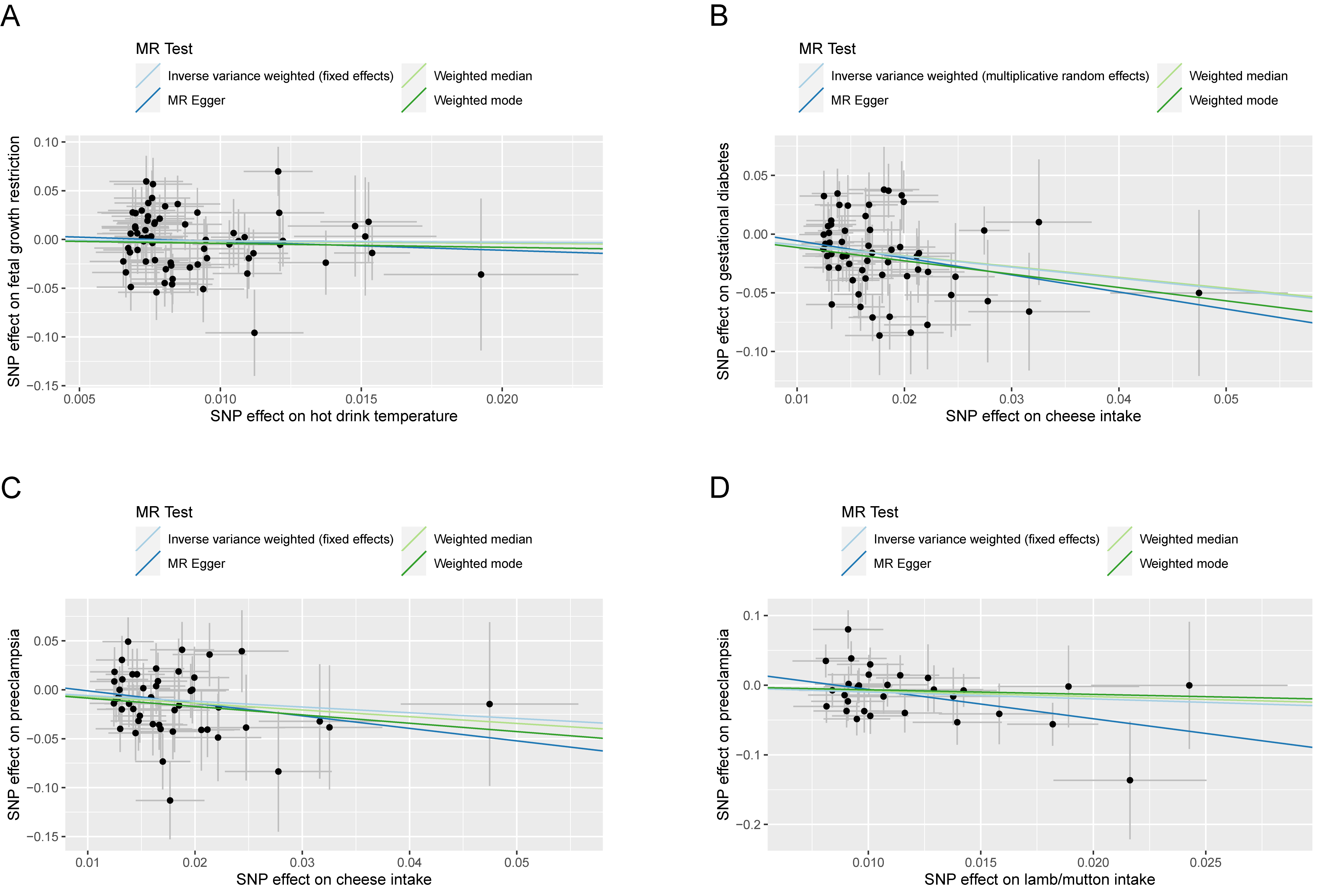

Supplement: Supplementary file 1 — Appendix S1. [file FSN3-12-8150-s001.zip › fsn34412-sup-0004-FigureS4.tif]
